# Estimating long‐term treatment effects in observational data: A comparison of the performance of different methods under real‐world uncertainty

**DOI:** 10.1002/sim.7664

**Published:** 2018-04-19

**Authors:** Simon J. Newsome, Ruth H. Keogh, Rhian M. Daniel

**Affiliations:** ^1^ Department of Medical Statistics London School of Hygiene and Tropical Medicine London UK; ^2^ Division of Population Medicine Cardiff University Cardiff UK

**Keywords:** causal inference, g‐computation formula, g‐estimation, inverse probability weighting, time‐dependent confounding

## Abstract

In the presence of time‐dependent confounding, there are several methods available to estimate treatment effects. With correctly specified models and appropriate structural assumptions, any of these methods could provide consistent effect estimates, but with real‐world data, all models will be misspecified and it is difficult to know if assumptions are violated.

In this paper, we investigate five methods: inverse probability weighting of marginal structural models, history‐adjusted marginal structural models, sequential conditional mean models, g‐computation formula, and g‐estimation of structural nested models. This work is motivated by an investigation of the effects of treatments in cystic fibrosis using the UK Cystic Fibrosis Registry data focussing on two outcomes: lung function (continuous outcome) and annual number of days receiving intravenous antibiotics (count outcome). We identified five features of this data that may affect the performance of the methods: misspecification of the causal null, long‐term treatment effects, effect modification by time‐varying covariates, misspecification of the direction of causal pathways, and censoring.

In simulation studies, under ideal settings, all five methods provide consistent estimates of the treatment effect with little difference between methods. However, all methods performed poorly under some settings, highlighting the importance of using appropriate methods based on the data available. Furthermore, with the count outcome, the issue of non‐collapsibility makes comparison between methods delivering marginal and conditional effects difficult. In many situations, we would recommend using more than one of the available methods for analysis, as if the effect estimates are very different, this would indicate potential issues with the analyses.

## 1 INTRODUCTION

Advanced methods for causal inference in longitudinal observational studies are an important tool for investigating treatment effects in nontrial settings where the presence of time‐dependent confounders generally precludes the use of simpler conventional methods. Time‐dependent confounding is an issue in longitudinal studies when a time‐varying covariate is affected by treatment, but this covariate then also subsequently affects the probability of receiving future treatment as well as affecting the outcome of interest.^1^

In these situations, there are a number of methods available to researchers, and one of these methods, in particular, inverse probability weighting (IPW) of marginal structural models (MSM), has become increasingly popular in applied research. The increasing use of this method over other methods may in part be due to its relative simplicity, but it is not clear if other methods may be better suited to some analyses. The methods investigated in this paper are motivated by questions about the efficacy of long‐term treatment use in cystic fibrosis (CF) and the challenges for addressing these using longitudinal observational data from a patient registry. In addition to IPW of MSM, we identified four other methods, which could be used in this setting: history‐adjusted marginal structural models (HA‐MSM), sequential conditional mean models (SCMM), g‐computation formula, and g‐estimation of structural nested models (SNM).

The primary aims of this paper are twofold: (1) to compare the ability and appropriateness of the different analysis methods for addressing the questions of interest, including to estimate treatment effect modification and to handle different outcome types and (2) to investigate the robustness of the different methods to handling practical challenges arising in longitudinal observational data, such as uncertainty about the relative temporality of measures and loss to follow‐up. In an ideal setting, where we could be sure that all assumptions are met and that all models are correctly specified, any one of the available methods could be used to obtain consistent treatment effect estimates. However, in reality, it can be difficult to know if assumptions hold and all models will be misspecified to some degree.

Section 2 of this paper gives more details about the UK CF Registry, the questions we wish to address, and specific details of the data that present challenges. This is followed in Section 3 by an overview of the five different methods, which we considered for the analysis of the Registry data. In Section 4, we present simulation studies investigating the performance of these five methods with two different types of outcomes (normally distributed continuous data and zero‐inflated negative binomial count data). An analysis of the UK CF registry data is presented in Section 5, and finally, we discuss the implications of the results of the simulation studies and data analysis in Section 6.

## 2 MOTIVATING EXAMPLE

### 2.1 CF and the UK Cystic Fibrosis Registry

Cystic fibrosis is the most common life‐threatening inherited disease in white people, and in the UK, there are over 10 000 people living with the disease.^2^, ^3^ Cystic fibrosis most seriously affects the lungs, where a build‐up in mucus causes breathing difficulties and leads to an increase in respiratory infections. There are now many treatments available that can help improve the health of people with CF, but many of these treatments are very time consuming and often treatments are not stopped once started. This leads to an accumulation of treatments, and treatment burden is a common complaint among people with CF.^4^

Almost all treatments currently used in CF care were approved following a successful clinical trial. However, a limitation of many trials is that they are short in duration, whereas in practice, treatments are used long term. In most cases, it would not be feasible to run trials for such long periods of times, and it could also be unethical to continue to withhold treatment from patients if a strong short‐term benefit has been observed.

The UK CF Registry is a national database, which has collected annual data on almost all people with CF in the UK since 2007. At an annual assessment, detailed information is obtained on many different measures of health status as well as all the treatments received in the past year.^5^

This paper will focus on one common CF treatment, dornase alfa (DNase), which was licensed for use in the UK in 1994 after a randomised trial showed efficacy at improving lung function over a 6‐month period.^6^ Subsequent studies have investigated the effects of up to 2 years' use of DNase, but in practice, patients generally continue to receive DNase indefinitely once treatment has been started.^7^

To illustrate the potential of the statistical methods with different types of outcomes, this paper will consider the effects of treatment on two important clinical outcomes: percent predicted forced expiratory volume in 1 second (ppFEV_1_) (a continuous outcome measuring an individual's lung function) and annual number of days of intravenous antibiotic therapy (IV days) (a count outcome of the number of days an individual received intravenous antibiotics in a given year). The decision to start prescribing DNase to a patient depends on many factors, including pretreatment measures of ppFEV_1_ and annual IV days, which are then in turn potentially affected by treatment use.

**Figure 1:**
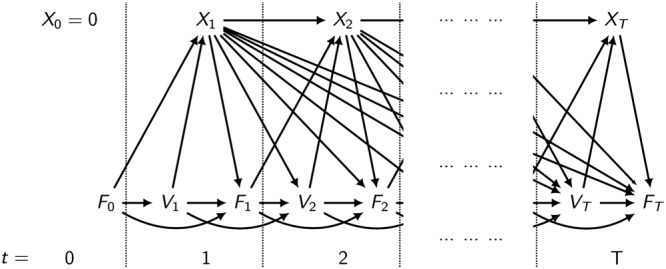
Directed acyclic graph of causal pathways between treatment (X_t_), ppFEV_1_ (F_t_), and IV days (V_t_). Baseline and other time‐varying confounders are not shown for clarity

Figure 1 shows a directed acyclic graph (DAG) of the assumed causal pathways between key variables in the UK CF Registry. Data are obtained at annual visits. Treatment status at visit *t* is denoted *X*_*t*_, *F*_*t*_ denotes ppFEV_1_, and *V*_*t*_ denotes IV days. We focus on patients not using treatment at a baseline visit 0, *X*_0_ = 0. At subsequent annual visits, their ppFEV_1_ on that day is recorded, and data about the previous year are also collected, such as which treatments they received throughout the year and their total number of IV days.

The DAG visualises ppFEV_1_ at visit *t*−1 affecting treatment at visit *t*, annual IV days at visit *t* affecting treatment at visit *t*, DNase use at visit *t* affecting all future measures of ppFEV_1_ and annual IV days, and direct longitudinal associations between ppFEV_1_ and annual IV days. There may also be other important baseline or time‐varying confounders, which have not been included in the DAG for clarity. In this paper, we focus on investigating the effect of treatment on lung function and IV days and how this effect might change with continued use of treatment over several years.

### 2.2 Features and challenges in the analysis of this data

As is common with most observational data, there are a number of issues that need to be considered when approaching the analysis of the UK CF Registry data.

One challenge is the use of the available methods with different types of outcomes. One of our outcomes of interest, ppFEV_1_, is continuous and can be approximated by a conditionally normal distribution. All of the methods described in this paper can easily accommodate such an outcome. However, the other outcome, annual IV days, is a count outcome ranging from 0 to 365, which we model with a zero‐inflated negative binomial distribution. This can be harder to incorporate into some of the methods, and we will discuss these issues in Section 3.7.

In addition to considering two different types of outcome, we have identified 5 key features of the analysis of the UK CF registry. The first three of these question the ability and appropriateness of the different analysis methods for estimating the treatment effect: whether there exists any treatment effect at all, whether there are only short‐term or also long‐term effects, and whether there is effect modification of the treatment effect by time‐varying covariates. The second category are challenges that may arise because of the nature of the data available to investigate the above questions. Here, we consider the issues of censoring and uncertainty of the direction of causal pathways between variables.

The following subsections give further details on each of these five issues.

#### 2.2.1 Causal null hypothesis

The DAG shown in Figure 1 shows a causal effect of treatment on the outcomes of interest. To date, randomised trials have demonstrated the efficacy of DNase treatment in improving ppFEV_1_, but no studies have yet shown a significant effect of the treatment on reducing the rate of IV days. Furthermore, in a nontrial setting, where, for example, adherence levels may not be as high as in clinical trials, the findings of a causal effect of treatment may not be replicated. For this reason, methods that benefit from a degree of robustness to model misspecification at the causal null would be attractive.

#### 2.2.2 Long‐term treatment effects

**Figure 2:**
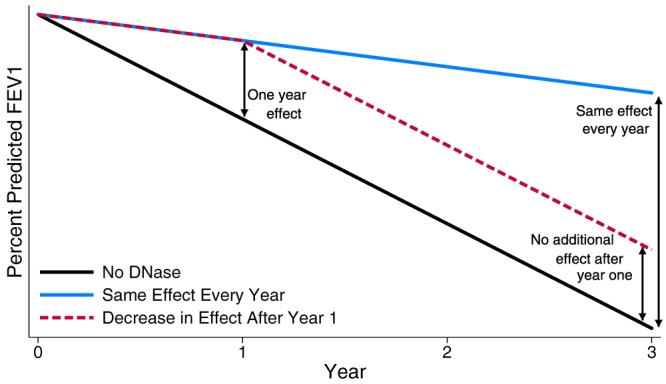
Two possible trajectories of lung function with long‐term dornase alfa treatment [Colour figure can be viewed at http://wileyonlinelibrary.com]

We define a long‐term treatment effect as an effect of *X*_*t*_ on *Y*_*s*_ (*s* > *t*) not mediated via intermediate treatments. No studies have previously looked at the effects of DNase beyond 2 years, and it therefore remains unknown how the effect of treatment might change with length of use. Taking the example of ppFEV_1_, two possible ways in which the treatment may affect the outcome are (1) the ppFEV_1_ trajectories of those receiving and not receiving treatment continue to grow apart indefinitely through time or (2) after the initial increase in ppFEV_1_ that has been observed at the start of taking treatment, the effectiveness of treatment may decrease with the two counterfactual trajectories no longer diverging. These two hypothetical lung function trajectories compared with the trajectory when not receiving treatment are shown in Figure 2. As it is unknown how the effect of treatment might change through time, it is important that the methods are flexible enough to identify the true long‐term effects.

#### 2.2.3 Effect modification by time‐varying covariates

We hypothesise that the effect of treatment may depend on the previous levels of ppFEV_1_ and number of IV days. This is because if a person starts treatment when they already have a healthy ppFEV_1_ level, it is unlikely that treatment could further improve ppFEV_1_, whereas it is realistic that the treatment could be much more effective in an individual with an lower ppFEV_1_. For informing practice, rather than just identifying the population average effect of treatment, it is important to gain understanding of how the effect of treatment might change depending on other covariates, and for this reason, it would be preferable to use a method that can test for the presence and estimate the strength of any effect modification.

#### 2.2.4 Misspecification of the direction of causal pathways

The DAG in Figure 1 includes assumptions about the direction of the causal pathways. For some variables, the appropriate direction of the causal pathway is clear (eg, the pathway from total IV days in 1 year to ppFEV_1_ measured at the end of the year). However, for other pathways, the appropriate direction for the arrow is less clear.

The direction of the causal pathway between treatment and number of IV days is particularly challenging. Both variables are summaries of the previous year, and some individuals may have had lots of IV days at the start of the year, which prompted them to start treatment, whereas others may have started treatment earlier, but then had IV days later in the year. In reality, therefore, the causal pathway between *X*_*t*_ and *V*_*t*_ is likely to go both ways, but in many methods, it will be necessary to specify just one direction for this pathway.

We have decided to focus on investigating the effect of *X*_*t*_ on *V*_*t*+1_, as, due to temporality, this pathway can only be directed this way, and to treat *V*_*t*_ as a confounder of this effect. In the real Registry data, we cannot know whether this is misspecified or not, and therefore, it is important to understand the potential extent of the bias in treatment effect estimates under different methods when the direction of this pathway has been misspecified.

#### 2.2.5 Censoring

We are fortunate that there are very few people lost to follow‐up in the UK CF Registry, and each year, there are relatively few deaths compared with the total number of people in the Registry. Nevertheless, it is possible that the fact that some individuals are censored for either of these reasons may bias the results. Therefore, we also wish to investigate how the different methods handle censoring. Although in reality there would likely be different processes affecting the probability that an individual dies or is lost to follow‐up, in this paper, we only consider one missing at random scenario where an individual's probability of being censored depends on previously measured variables.

## 3 METHODS

### 3.1 Notation and assumptions

We discuss the statistical methods with generic notation. Consider a cohort followed up annually from visit *t*=0 up to visit *t*=*T*. The treatment received at time *t* is denoted *X*_*t*_, and each year, a person can receive (*X*_*t*_=1) or not receive (*X*_*t*_=0) treatment during the period since the last visit. The outcome of interest, *Y*_*t*_, is also measured annually. We assume that at each visit, *X*_*t*_ precedes *Y*_*t*_ and define a 1‐year treatment effect to be the effect of *X*_*t*_ on *Y*_*t*_. We also have baseline confounders, **B**, and time‐varying confounders, **C**.

${\bar{X}}_{t}$ is a vector of the treatment history for an individual from visit 0 up to and including visit *t*, and we use the counterfactual notation $Y_{t}^{{\bar{x}}_{t}=\bar{1}}$ to refer to the outcome that would have been observed at visit *t* if an individual had received treatment up to and including visit *t*.

For all methods, we make the following four assumptions: no interference, positivity, consistency, and no unmeasured confounding. No interference means that for a given individual, their counterfactual outcome $Y_{t}^{\bar{x}}$ is not affected by the treatment that another individual receives.^8^ Positivity means that all individuals had a conditional probability strictly greater than 0 and strictly less than 1 of receiving treatment at all visits given their history, $0\;<\;P(X_{t}=1|{\bar{X}}_{t-1},{\bar{Y}}_{t-1},{\bar{C}}_{t},B)\;<\;1$.^9^ Consistency means that for each individual, the counterfactual outcome under the observed treatment is equal to the observed outcome, $Y_{i}=Y_{i}^{x_{i}}\;\text{when}\;x_{i}=X_{i}$.^10^ Finally, no unmeasured confounding means that conditional on the past observed variables the treatment received at visit *t* is independent of the counterfactual outcome, $Y_{t}^{{\bar{x}}_{t}}⫫\;X_{t}|{\bar{X}}_{t-1},{\bar{Y}}_{t-1},{\bar{C}}_{t},B$. 1

The following subsections give an overview of the methods that are considered for the analysis of the UK CF Registry. We introduce the methods with a continuous outcome in mind. Referring back to our motivating example and the DAG in Figure 1, we can consider ppFEV_1_(*F*_*t*_) to be the outcome of interest with IV days (*V*_*t*_) acting as a time‐dependent confounder. The count variable of IV days is also of interest as an outcome, and in Section 3.7, we outline how the methods can be extended for use with a count outcome.

### 3.2 IPW of marginal structural models

Inverse probability weighting of MSM^11^ has become an increasingly popular method to deal with time‐dependent confounding. We consider MSM of the following form:

(1) $$E\left(Y_{t}^{{\bar{x}}_{t}}\right)=\beta_{0}+\sum_{i=1}^{t}\beta_{x_{i}}x_{i},$$

where the $\beta_{x_{1}}$ to $\beta_{x_{t}}$ represent separate effects for treatment at each visit, thereby allowing for long‐term treatment effects. However, due to confounding, directly using the observed values and calculating $E[Y_{t}|{\bar{X}}_{t}={\bar{x}}_{t}]$ does not equate to the counterfactual $E[Y_{t}^{{\bar{x}}_{t}}]$.

Inverse probability weighting of the observations enables consistent estimation from an MSM by reweighting observations so that the levels of confounding variables become equally balanced between treated and untreated individuals. This is achieved by assigning large weights to individuals who were estimated to be unlikely to receive the treatment they actually received and downweighting observations for which there are lots of observations estimated to have similar propensities to receive the same treatment history.

To calculate the weights, one first estimates the propensity score, which is the probability of receiving treatment at each visit:

(2) $$P\left(X_{t}=1|{\bar{X}}_{t-1},{\bar{Y}}_{t-1},{\bar{C}}_{t},B\right)=\text{expit}\left(\beta_{0}+\beta_{X}X_{t-1}+\beta_{Y}Y_{t-1}+\beta_{C}C_{t}+\beta_{B}B\right).$$

Then this model is used to calculate the estimated probability that each person received the treatment they actually received, ie, for those who did receive treatment, we use the estimated probability from the above model and for those who did not receive treatment, 1 minus the estimated probability. The probability of their treatment history is then the product of these estimated probabilities from visit 1 up to visit *t*.

The inverse of the estimated probabilities can be used directly as the weights, but it is usually preferable to use so‐called stabilised weights^1^ where the numerator of the weights is the probability of receiving treatment based on previous treatment history and baseline covariates only,

(3) $$SW_{t}=\frac{P\left({\bar{X}}_{t}|{\bar{X}}_{t-1},B\right)}{P\left({\bar{X}}_{t}|{\bar{X}}_{t-1},{\bar{Y}}_{t-1},{\bar{C}}_{t},B\right)}=\prod_{i=1}^{t}\frac{P\left(X_{i}|{\bar{X}}_{i-1},B\right)}{P\left(X_{i}|{\bar{X}}_{i-1},{\bar{Y}}_{i-1},{\bar{C}}_{i},B\right)}.$$

A final MSM, such as that given in Equation 1, can then be fit where the observations are weighted using the estimated weights. However, note that any baseline confounders included in the numerator of Equation 3 must also be included in the MSM. This would result in a conditional estimate, meaning if a marginal estimate is desired then no confounders should be included in the numerator.

Due to the fact that time‐varying covariates are not included in the MSM, this method does not allow for the estimation of effect modification by time‐varying covariates. However, the method also does not need the assumption that there is no effect modification and will estimate consistent population average effects even if the effect of treatment is not uniform for the whole population.

Using stabilised weights helps to reduce the variability in the weights, but in cases where there are strong time‐varying predictors of treatment, the weights can remain highly variable that can lead to instability. Therefore, it can sometimes be preferable to truncate the most extreme weights, even though this may introduce some bias.^12^, ^13^ In this paper, we will present the results of IPW analyses with and without truncation of the stabilised weights to the 1^st^ and 99^th^ percentile.

In the presence of censoring, it is also possible to incorporate censoring weights into the analysis. Similarly to the previously described weights, we weight individuals with stabilised inverse weights of their estimated probability of being censored before visit *t*,

(4) $$\text{LTFU}W_{t}=\prod_{i=1}^{t}\frac{P\left({\text{LTFU}}_{i}\right)}{P\left({\text{LTFU}}_{i}|{\bar{X}}_{i-1},{\bar{Y}}_{i-1},{\bar{C}}_{i-1},B\right)}.$$

Using this method, we assume that future visits are missing at random, ie, censoring is affected by previously measured variables. The estimated censoring weights can then be multiplied by the estimated stabilised weights to give the weights to be used to account for bias due to both confounding and censoring.

### 3.3 History‐adjusted marginal structural models

As stated in the previous section, one limitation of IPW of MSM is that effect modification of the treatment effect by time‐varying covariates cannot be estimated. Therefore, in cases where the estimation of an interaction term is desired, HA‐MSM are an extension to IPW of MSM, which do allow for this.^14^

In the standard MSM described in the Section 3.2, observations are reweighted based on all covariates measured after baseline until the visit of the outcome of interest. In an HA‐MSM, the reweighting is done separately from each time of treatment *t* up to the time of outcome, *s* (*t* ≤ *s*). Covariates measured prior to the treatment at time *t* can be included in the final HA‐MSM in the same way as baseline covariates were included in the standard MSM.

Formally, the stabilised weights for exposure at time *t* on outcome at time *s* are given by

(5) $$SW_{ts}=\prod_{i=t}^{s}\frac{P\left(X_{i}|{\bar{X}}_{i-1},B\right)}{P\left(X_{i}|{\bar{X}}_{i-1},{\bar{Y}}_{i-1},{\bar{C}}_{i},B\right)},$$

and an example of the HA‐MSM could be given by

(6) $$E\left(Y_{s}^{{\bar{x}}_{s}}|{\bar{x}}_{t-1},{\bar{y}}_{t-1},{\bar{c}}_{t},b\right)=\beta_{0}+\beta_{b}b+\beta_{c}c_{t}+\beta_{x}x_{t-1}+\beta_{y}y_{t-1}+\sum_{i=t}^{s}\beta_{x_{i}}x_{i}+\sum_{i=t}^{s}\beta_{{\text{int}}_{i}}x_{i}y_{t-1}.$$

In Equation 6, we have included an interaction term between previous measures of the outcome (itself a time‐varying confounder) and treatment so as to allow the estimation of any effect modification.

As with IPW of MSM, it is also possible to estimate censoring weights, in this case estimating an individual's probability of being censored between visits *t* and *s* and multiplying these weights with the stabilised weights.

### 3.4 Sequential conditional mean models

Even in the presence of time‐dependent confounding, it is still possible to use standard regression methods, but these methods can only estimate total effects.^15^ The total effect of a treatment, *X*_*t*_, on an outcome *Y*_*s*_ (*s*>*t*) would include not only the direct effect of *X*_*t*_ on *Y*_*s*_ and the indirect effects of *X*_*t*_ on *Y*_*s*_ through time‐varying covariates but also the indirect effect of *X*_*t*_ on *Y*_*s*_ mediated through future exposures. It cannot, therefore, be used to investigate the effect of receiving 2 years' treatment in our example, as some people discontinue treatment. For this reason, in the examples here, this method is only used to estimate “short‐term” effects, which we define as the effect of 1‐year treatment on the outcome measured at the end of the year.

These SCMM will give a consistent estimate of the 1‐year effect of treatment as long as we appropriately control for all confounding effects of this short‐term effect. For example, the following short‐term model would suffice if the most recent measures of all covariates were sufficient to remove confounding:

(7) $$E\left(Y_{t}|{\bar{X}}_{t},{\bar{Y}}_{t-1},{\bar{C}}_{t},B\right)=\beta_{0}+\beta_{X_{1}}X_{t}+\beta_{X_{2}}X_{t-1}+\beta_{Y}Y_{t-1}+\beta_{C}C_{t}+\beta_{B}B.$$

It is also possible to incorporate propensity scores into the SCMM to provide a doubly robust estimator. The propensity score can be calculated as it was in the IPW method by using Equation 2, and this is then incorporated into the SCMM as follows:

(8) $$E\left(Y_{t}|{\bar{X}}_{t},{\bar{Y}}_{t-1},{\bar{C}}_{t},B,p_{t}\right)=\beta_{0}+\beta_{X_{1}}X_{t}+\beta_{X_{2}}X_{t-1}+\beta_{Y}Y_{t-1}+\beta_{C}C_{t}+\beta_{B}B+\beta_{p}p_{t}.$$

Although this method cannot provide estimates for the effects of varying lengths of treatment duration, the simplicity of the method is appealing, and these short‐term effect estimates can also be compared with the 1‐year treatment effect estimates from the other methods. SCMM also form the first step of the next 2 methods: g‐computation formula and g‐estimation of SNM.

### 3.5 G‐computation formula

The g‐computation formula first described by Robins^16^ is another method that can deal with the issue of time‐dependent confounding to give consistent estimates of long‐term treatment effects. In this method, short‐term models, ie, models for 1‐year time effects, for all time‐varying covariates (in our example, *Y* and **C**) are used to simulate counterfactual outcomes under different treatment trajectories sequentially through time.

For example, the time‐varying continuous outcome *Y* could be modelled by Equation 7 and counterfactuals for *Y*_1_ could then be simulated setting everyone either receiving or not receiving treatment at visit 1:

(9) $${\tilde{Y}}_{1}^{x_{1}=1}={\hat{\beta }}_{0}+{\hat{\beta }}_{Y}Y_{0}+{\hat{\beta }}_{C}C_{1}+{\hat{\beta }}_{B}B+{\hat{\beta }}_{X_{1}}+\tilde{\varepsilon },$$

(10) $${\tilde{Y}}_{1}^{x_{1}=0}={\hat{\beta }}_{0}+{\hat{\beta }}_{Y}Y_{0}+{\hat{\beta }}_{C}C_{1}+{\hat{\beta }}_{B}B+\tilde{\varepsilon },$$

where $\tilde{\varepsilon }$ is a random draw from a normal distribution whose standard deviation is the model‐estimated root mean square error, resulting in simulated counterfactual measures.

Similar short‐term models would need to be specified for all time‐varying covariates **C** to allow the counterfactuals for all covariates to be simulated at visit 1. In our example, we have one time‐varying confounder, which follows a zero‐inflated negative binomial distribution. Therefore, this was used to model the data and then to simulate random draws for the count for each individual.

The process can then be repeated sequentially for all visits. For example, at visit 2, there would be four counterfactuals simulated for each individual, corresponding to (1) receiving treatment at both visits, (2) at the first visit only, (3) at the second visit only, or (4) never receiving treatment. These counterfactuals could be simulated, respectively, as follows:

(11) $${\tilde{Y}}_{2}^{x_{1}=1,x_{2}=1}={\hat{\beta }}_{0}+{\hat{\beta }}_{Y}{\tilde{Y}}_{1}^{x_{1}=1}+{\hat{\beta }}_{C}{\tilde{C}}_{2}^{x_{1}=1}+{\hat{\beta }}_{B}B+{\hat{\beta }}_{X_{1}}+{\hat{\beta }}_{X_{2}}+\tilde{\varepsilon },$$

(12) $${\tilde{Y}}_{2}^{x_{1}=1,x_{2}=0}={\hat{\beta }}_{0}+{\hat{\beta }}_{Y}{\tilde{Y}}_{1}^{x_{1}=1}+{\hat{\beta }}_{C}{\tilde{C}}_{2}^{x_{1}=1}+{\hat{\beta }}_{B}B+{\hat{\beta }}_{X_{2}}+\tilde{\varepsilon },$$

(13) $${\tilde{Y}}_{2}^{x_{1}=0,x_{2}=1}={\hat{\beta }}_{0}+{\hat{\beta }}_{Y}{\tilde{Y}}_{1}^{x_{1}=0}+{\hat{\beta }}_{C}{\tilde{C}}_{2}^{x_{1}=0}+{\hat{\beta }}_{B}B+{\hat{\beta }}_{X_{1}}+\tilde{\varepsilon },$$

(14) $${\tilde{Y}}_{2}^{x_{1}=0,x_{2}=0}={\hat{\beta }}_{0}+{\hat{\beta }}_{Y}{\tilde{Y}}_{1}^{x_{1}=0}+{\hat{\beta }}_{C}{\tilde{C}}_{2}^{x_{1}=0}+{\hat{\beta }}_{B}B+\tilde{\varepsilon }.$$

The counterfactual outcomes under different treatment trajectories can then be compared with a MSM, eg,

(15) $$E\left(Y_{t}^{{\bar{x}}_{t}}\right)=\beta_{0}+\sum_{i=1}^{t}\beta_{x_{i}}x_{i}.$$

One well‐known drawback of the use of this method with non‐linear models is the g‐null paradox.^16^, ^17^ This is an issue whereby given a large enough sample size, the causal null hypothesis will always be rejected even if there is in fact no treatment effect. This is due to the fact that the combination of different parametric models will be inconsistent with the null hypothesis.

### 3.6 G‐estimation of structural nested models

The final method we will consider is g‐estimation of SNM.^18^ This method has been used less than the previously described methods, and this may partly be due to the perceived difficulty of applying the method with standard statistical software.^19^ However, a recent paper by Vansteelandt and Sjolander revisits g‐estimation, showing how it can be implemented with standard software.^20^

Similar to HA‐MSM, this method can estimate the effect of all treatments at visits *t* on outcomes at visits *s* where *t*≤*s*. Starting from the short‐term model as in the SCMM, we obtain an estimate for the 1‐year effect of treatment, $\beta_{X_{1}}$,

(16) $$E\left(Y_{t}|{\bar{X}}_{t},{\bar{Y}}_{t-1},{\bar{C}}_{t},B,p_{t}\right)=\beta_{0}+\beta_{1}X_{t-1}+\beta_{2}Y_{t-1}+\beta_{3}C_{t}+\beta_{4}B+\beta_{5}p_{t}+\beta_{X_{1}}X_{t},$$

where *p*_*t*_ is the estimated propensity score.

The estimate $\beta_{X_{1}}$ can then be used to construct counterfactuals by subtracting the estimated 1‐year effect to be able to see if there is any extra effect for additional years of treatment,

(17) $$H_{st}=Y_{s}-\sum_{u=t+1}^{s}\beta_{X_{s-u+1}}X_{u}.$$

It can be seen that in the case where *t* = *s*, *H*_*st*_ is simply equal to *Y*_*s*_ as expected, whereas intermediate treatment effects are subtracted if *t* < *s*. In the first iteration, as we only have an estimate for $\beta_{X_{1}}$, we can only calculate *H*_*st*_ where *s* ≤ *t* + 1. However, this now allows us to estimate both the 1‐year and 2‐year effects with the following model:

(18) $$E\left(H_{sj}|{\bar{X}}_{j},{\bar{Y}}_{j-1},{\bar{C}}_{j},B,p_{j}\right)=\beta_{0}+\beta_{1}X_{j-1}+\beta_{2}Y_{j-1}+\beta_{3}C_{j}+\beta_{4}B+\beta_{5}p_{j}+\beta_{X_{s-j+1}}X_{j}.$$

Iteration of Equations 17 and 18 allows the estimation of all $\beta_{X_{s-j+1}}$ where 1 ≤*j* ≤ *s* and 1 ≤ *s* ≤ *T*.

Similarly to HA‐MSM, g‐estimation is a method that allows the estimation of effect modification by time‐varying covariates by including interaction terms in both Equations 17 and 18.

Censoring weights as described in Section 3.3 can also be incorporated into g‐estimation, weighting individuals by their estimated probability of being censored between visits *t* and *s*.

### 3.7 Use of methods with a count outcome

For our motivating example, we have 2 outcomes of interest, ppFEV_1_ and annual IV days. The first of these is a continuous outcome, and all 5 of the methods can easily handle this outcome. More care is needed when considering annual IV days, which is a count outcome ranging from 0 to 365.

Upon investigation, IV days can be considered approximately distributed by a zero‐inflated negative binomial distribution. Modelling this outcome therefore requires two separate estimation procedures: (1) logistic regression to estimate the odds of a count of zero IV days and (2) negative binomial regression to estimate the rate of IV days. Therefore, there are two separate parts to the treatment effect: the estimated effect of treatment on having zero IV days (an odds ratio) and the estimated effect of treatment on the number of IV days (a rate ratio).

For SCMM, this is not an issue, as one can simply fit a zero‐inflated negative binomial model to estimate both effects. Similarly, IPW, HA‐MSM, and g‐computation formula can all handle different types of outcome by just changing the final MSM, eg,

(19) $$E\left(Y_{t}^{{\bar{x}}_{t}}\right)=\text{expit}\left(\beta_{0}+\sum_{i=1}^{t}\beta_{x_{i}}x_{i}\right)\exp\left(\gamma_{0}+\sum_{i=1}^{t}\gamma_{x_{i}}x_{i}\right).$$

Unlike the other 4 methods, which can easily handle different types of outcome, the method of g‐estimation described in Section 3.6 has until recently only been described for continuous outcomes. However, a recent paper has shown how this method can be adapted to allow for a count outcome by modelling with a gamma distribution.^21^ This allows for the estimation of the effect of treatment on the rate of IV days, but would still not allow for the decomposition of the effect into the probability of a zero count and a rate, as the other methods do.

Another issue one needs to consider when modelling a count outcome is non‐collapsibility. Unlike with the continuous outcome where, thanks to collapsibility, the marginal and conditional effects are the same, this no longer holds for models suitable for count outcomes. Thus, the treatment effect on IV days will differ between methods depending on whether the method delivers a marginal effect (IPW and g‐computation formula) or a conditional effect (SCMM, HA‐MSM and g‐estimation).

### 3.8 Overview of methods

Referring back to the five features of the UK CF Registry introduced in Sections 2.2.1 to 2.2.5, we would hope for any method to estimate no treatment effect on average when there is no treatment effect, but the g‐null paradox may mean that the g‐computation formula could perform poorly in this setting.

Except for SCMM, all methods can estimate long‐term treatment effects, and in all our analyses, we will include separate terms for treatment at each visit making no assumptions about a continuous effect or a trend effect. SCMM will only be used to estimate the short‐term treatment effect, but the method will consistently estimate this even if there are longer term effects.^15^

In terms of effect modification, three of the methods (SCMM, g‐estimation, and HA‐MSM) allow interaction terms, meaning that when this is of interest, only these methods can be used. IPW of MSM and g‐computation formula can still be used to estimate population average effects even in the presence of effect modification by time‐varying covariates, whereas the other three methods may show bias in estimating population average effects if there is in fact effect modification and it is not explicitly modelled.

When there is censoring, three of the methods (IPW of MSM, HA‐MSM, and g‐estimation) can use censoring weights to correct for the individuals who do not have full follow‐up. Censoring should not affect the short‐term models used in SCMM, and similarly, the g‐computation formula uses the same short‐term models and then simulates follow‐up without censoring.

With the exception of SCMM, it is normally advised to use a bootstrap procedure to obtain standard errors (SE). This is because all the methods contain a number of steps of estimation and just using the final model‐based SE would fail to account for the uncertainty from the earlier steps.^13^, ^14^, ^22^, ^23^ The bootstrap provides valid results as all of these methods produce regular estimators.^24^

## 4 SIMULATION STUDIES

The following section gives details of simulation studies that were performed to investigate how the features and challenges identified in Section 2.2 (robustness to misspecification of the causal null, long‐term treatment effects, effect modification by time‐varying covariates, misspecification of the direction of causal pathways, and censoring) affect the performance of the five methods given in Section 3 (SCMM, IPW of MSM, HA‐MSM, g‐computation formula, and g‐estimation of SNM).

The aims of the simulation studies are to understand how the performance of the analysis methods might be affected by these challenges and to help provide a framework for the best analysis strategy for the real UK CF Registry data. The simulation studies were performed following the guidelines given by Burton et al^25^ and full details of the design of the simulation studies can be found in Supporting Information, with a summary below.

### 4.1 Design of simulation studies

**Figure 3:**
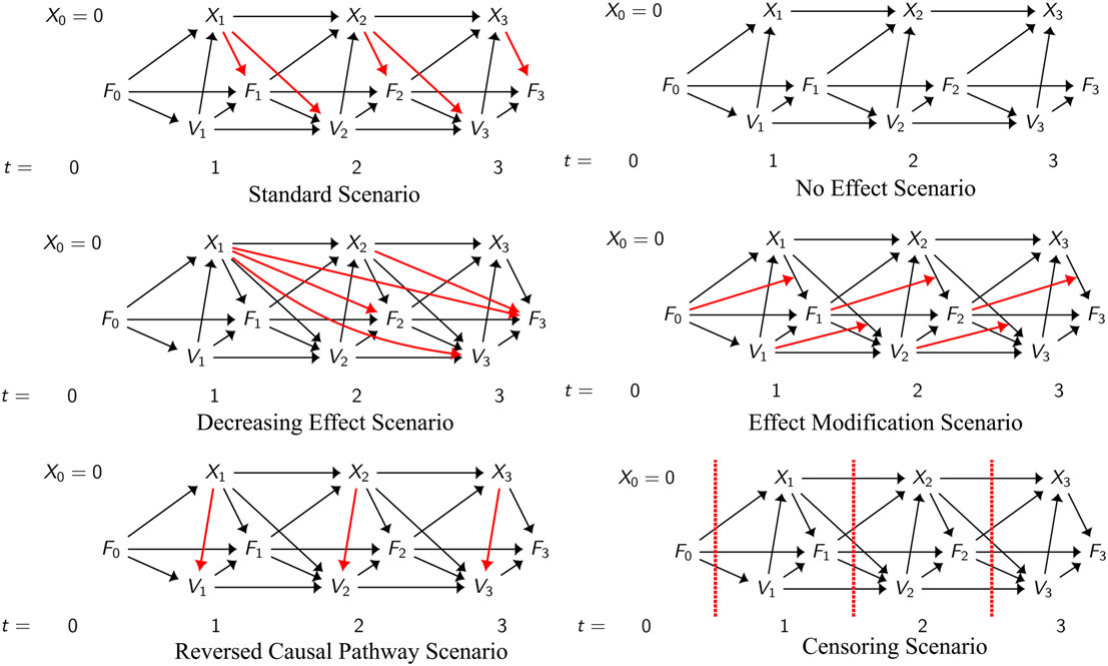
Simplified directed acyclic graphs showing data generation process for each of the 6 scenarios investigated. The real data were generated for up to 5 visits, and there is additionally a baseline confounder, age, affecting all variables

Datasets were simulated for six different scenarios as shown in Figure 3. In each DAG, the arrows highlighted in red show the specific differences compared with the other scenarios.

The first scenario is the standard scenario, which will be the baseline with which to compare the other methods. In this scenario, there is a 1‐year treatment effect, there are no direct long‐term effects, although there are long‐term effects mediated through other time‐varying covariates. This is the scenario for which all the methods will be correctly specified and as such we would expect all methods to provide consistent estimates for the treatment effects in this scenario.

In the second scenario, we simulate without any treatment effect, and the third scenario adds long‐term direct effects of treatment. In this case, the long‐term direct effects are actually negative effects, slightly counteracting the beneficial 1‐year effects, resulting in a decrease in the treatment effect through time.

The fourth scenario simulates effect modification of treatment by time‐varying covariates. Although effect modification is generally not shown in DAGs, we have included arrows in Figure 3 to help illustrate how the presence of effect modification would change the treatment effect.

The fifth scenario concerns the direction of the causal pathway between treatment and IV days in the same year. In our analysis, we will always analyse the data as if the direction of the causal pathway is from *V*_*t*_ to *X*_*t*_, even when the data have actually been simulated the other way around, ie, *X*_*t*_ affects *V*_*t*_.

In the final scenario, individuals can either all be followed up for 5 visits, or there can be some censoring, whereby “unhealthy” individuals are more likely to be censored at an earlier visit. This corresponds to a missing at random scenario, whereby the probability of being censored depends on observed variables.

For each scenario, we simulated 1000 datasets, each with 7500 individuals. The data were generated so as to imitate the observed data in the Registry as closely as possible. In the real data, there are many treatments that individuals could be receiving and also many covariates that might be confounders. For the simulation studies, we kept just one binary treatment, *X*_*t*_, and the 2 outcome variables, ppFEV_1_(*F*_*t*_) and annual IV days (*V*_*t*_), which also act as time‐dependent confounders. Lung function was simulated as a continuous variable with a normal distribution and IV days as a count outcome following a zero‐inflated negative binomial distribution. In addition to these 3 variables, we also generated age from a beta distribution corresponding to what was observed in the real data to act as a baseline confounder.

For each method and each scenario, we run two analyses: the first considering ppFEV_1_ as the outcome and the second annual IV days as the outcome. For each simulation, the coefficients corresponding to the treatment effects will be stored. For SCMM, which can only measure short‐term effects, only the coefficient corresponding to 1 year of treatment will be stored. For all other methods, we estimate the effects of up to 5 years' treatment use on ppFEV_1_ and up to 4 years' treatment use on IV days. The reason for this difference is due to the 1‐year effect of treatment on lung function being defined as $\bar{X_{t}}\to F_{t}$, whereas the 1‐year effect of treatment on IV days is $\bar{X_{t}}\to V_{t+1}$. As such, there is always one extra year of data available for the lung function outcome.

We will compare the methods based on the bias, empirical SE, and mean squared error (MSE). Although it is known that the model‐based SE are biased for most of these methods, we will also store the estimated robust SE so as to compare them to the empirical SE.

### 4.2 Results of simulation studies

#### 4.2.1 Continuous outcome

**Figure 4:**
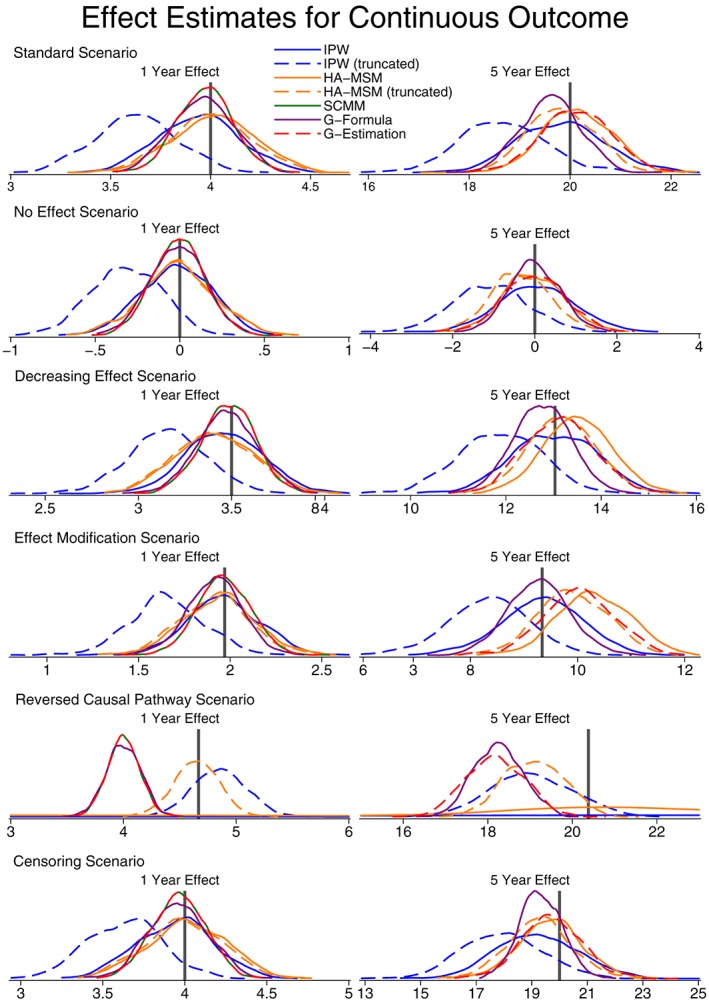
Kernel density plots showing the distribution of population‐average effect estimates for a continuous outcome. The vertical line shows the correct effect. HA‐MSM, history‐adjusted marginal structural models; IPW, inverse probability weighting; SCMM, sequential conditional mean models

In Figure 4, we present kernel density plots showing the results of the simulation studies for the normally distributed continuous outcome. We only present results for the 1‐year effect and the 5‐year effect to show the 2 extremes of short‐ to long‐term effects. In all cases, the results for the 2‐ to 4‐year effects followed the trend between the 1‐year and 5‐year effects. More details of the results can be found in Table S3.

As expected, all 5 methods to provide consistent estimates for the “standard” scenario where all the models are correctly specified. The only method that performs poorly here is using truncation with IPW, but this is also to be expected as it is known that due to truncation the weights would no longer fully account for confounding. The 5‐year treatment effect estimates are slightly biased, but when using a much larger sample size, all methods were unbiased; therefore, we believe this residual bias is due to the sample size, which we have kept at 7500 individuals as it is unlikely that we would ever obtain a larger sample from the UK CF Registry.

These findings are repeated for the scenarios where there is no treatment effect, where the treatment effect decreases over time, and where there is censoring (provided that censoring weights are used for IPW, HA‐MSM, and g‐estimation).

For the scenario where the causal pathway between a confounder and treatment is specified the wrong way round, we find that the situation is the opposite: All methods are biased, but IPW and HA‐MSM perform comparatively well when the weights are truncated. However, untruncated, they perform very poorly with very large variability and even fail to converge on an estimate many times.

When considering effect modification by time‐varying covariates, all the methods can still be used to provide an estimate for the population‐average effect. For the 1‐year effects, all the methods provided consistent estimates; however, at 5 years, there was some noticeable bias for g‐estimation and HA‐MSM. These are the 2 methods that can incorporate the estimation of effect modification by time‐varying covariates, and not including these interactions terms when they are in fact present has introduced bias. Conversely, although IPW and g‐computation formula cannot estimate interaction terms, they do not assume that there is no effect modification and can provide consistent estimates for the population‐average effect.

**Figure 5:**
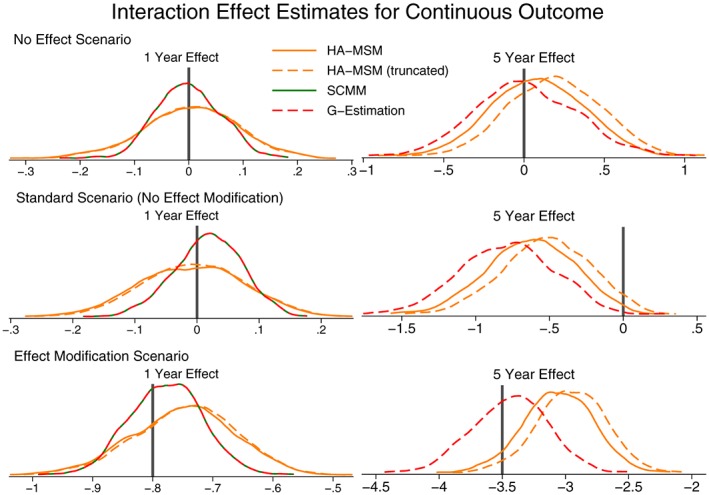
Kernel density plots showing the distribution of interaction effect estimates for a continuous outcome. The vertical line shows the correct effect. HA‐MSM, history‐adjusted marginal structural models; IPW, inverse probability weighting; SCMM, sequential conditional mean models

If the aim is to estimate the strength of any effect modification by time‐varying covariates, then it would be necessary to use HA‐MSM, SCMM, or g‐estimation, and these results are presented in Figure 5 (and Table S4). We see that all 3 methods perform similarly well in estimating interaction terms, although there is still some finite sample size bias, and a much larger sample size would be needed to accurately estimate the interaction terms. Even in cases where there is no effect modification, including an interaction term in the models did not introduce bias, and the methods correctly estimate zero for the interaction term on average.

When considering the SE, only in SCMM and HA‐MSM did the model‐estimated SE approximate the empirical SE. This is theoretically known in the case of SCMM with the propensity score known and, therefore, will be approximately correct when the propensity score is well estimated. In the case of HA‐MSM, we believe this to be a peculiarity of our simulation setting, and it is unlikely to be true generally. For this reason, for all methods other than SCMM, a bootstrap procedure should be used to obtain reliable SE estimates. Comparing the methods, g‐computation formula consistently shows the smallest empirical SE, followed by SCMM, g‐estimation, and HA‐MSM with similar SE and, finally, IPW with the largest SE. In the scenario of reversed causal pathways, IPW and HA‐MSM had especially large SE when untruncated weights were used.

#### 4.2.2 Count outcome

**Figure 6:**
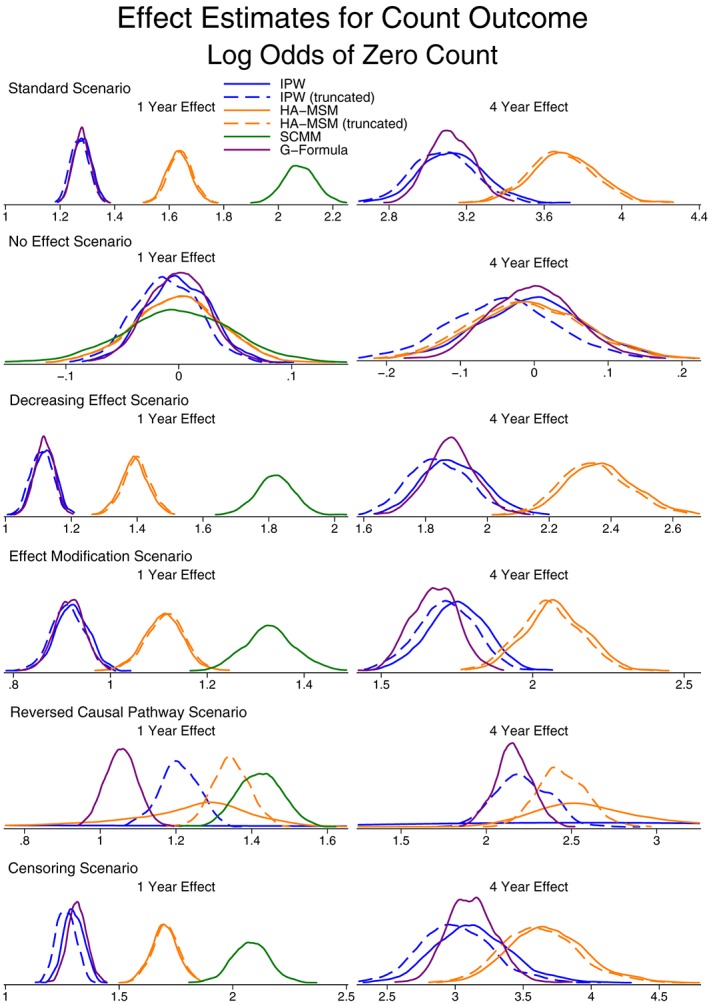
Kernel density plots showing the distribution of population‐average effect estimates for the odds of a zero count. HA‐MSM, history‐adjusted marginal structural models; IPW, inverse probability weighting; SCMM, sequential conditional mean models

**Figure 7:**
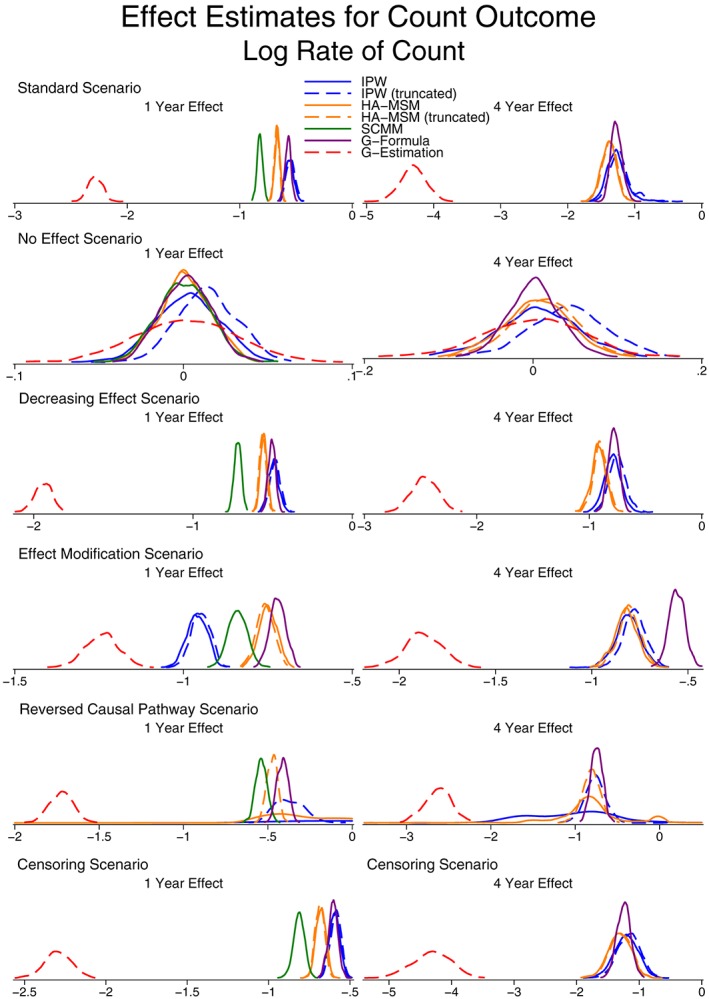
Kernel density plots showing the distribution of population‐average effect estimates for the rate of a count outcome. HA‐MSM, history‐adjusted marginal structural models; IPW, inverse probability weighting; SCMM, sequential conditional mean models

Unlike with the continuous outcome, due to the issue of non‐collapsibility, we do not compare the effect estimates for the count outcome to a “correct” value. However, Figures 6 and 7 present the effect estimates and SE for both the odds of a zero count and the rate of the count. As with the continuous outcome, more detailed results can be found in Table S5.

Both IPW and g‐computation formula provide marginal effect estimates and in almost all cases provide very similar estimates. The only setting where they do not provide similar estimates is the case of reversed causal pathways where IPW performs very poorly with very large variability, as was also seen for the continuous outcome.

Considering the 3 methods that provide conditional effect estimates, we note that the methods are not in general in agreement, and this is due to the fact that the final models condition on different subsets of variables. In the case of g‐estimation, due to the fact that the method can only estimate a rate (rather than also accounting for the separate process of excess zeroes), the estimates from this method are generally very different from all other methods.

The only case where all 5 methods are in agreement is when there is no treatment effect. Here, both the marginal and conditional effect estimates are zero. This suggests that any method could be used to perform a test of the null hypothesis of no treatment effect, but the strength of any effect estimates cannot directly be compared between methods.

**Figure 8:**
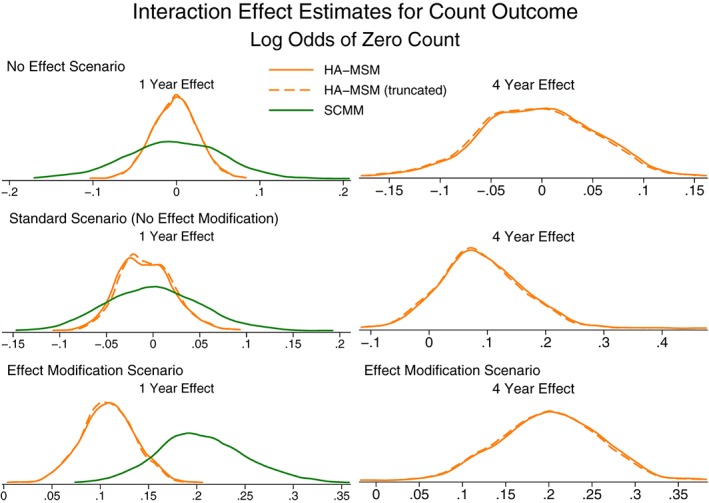
Kernel density plots showing the distribution of interaction effect estimates for the odds of a zero count. HA‐MSM, history‐adjusted marginal structural models; SCMM, sequential conditional mean models

**Figure 9:**
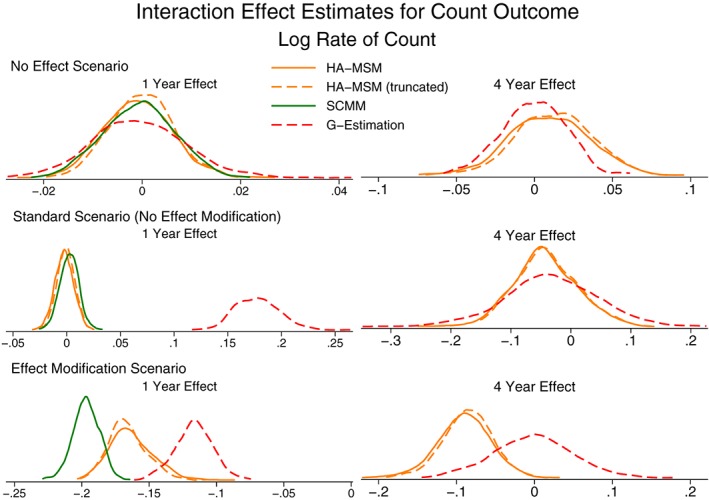
Kernel density plots showing the distribution of interaction effect estimates for the rate of a count outcome. HA‐MSM, history‐adjusted marginal structural models; SCMM, sequential conditional mean models

The results for estimating interaction terms are presented in Figures 8 and 9 (Table S6). The findings are similar to the case of interaction terms with continuous outcomes, except for the case of g‐estimation where even in the case where there is no effect modification in the data generation process, the method did not average on no effect modification. This is again due to non‐collapsibility, where although there is no effect modification present when assuming the data follow a zero‐inflated negative binomial distribution, there may be under different distributional models.

Similar to continuous outcomes, the model estimated SE from HA‐MSM and SCMM approximated the empirical SE well, but again, we would recommend a bootstrap procedure to be used for all methods other than SCMM.

## 5 DATA ANALYSIS

Based on the findings from the simulation studies, if the real causal pathways in the Registry data are similar to those used in the simulation studies, all 5 available statistical methods would be suitable to investigate the effects of DNase on ppFEV_1_ and annual number of IV days.

Unfortunately, the key challenge identified in the simulation studies was that misspecifying the direction of a causal pathway will introduce bias no matter which method is used. In the Registry data, it is likely that the real direction of the causal pathway between treatment (*X*_*t*_) and annual IV days (*V*_*t*_) is somewhere between the two extremes of the best‐case scenario where *X*_*t*_ is only affected by *V*_*t*_ and the worse‐case scenario where *X*_*t*_ only affects *V*_*t*_. In this setting, the simulation studies showed that we might expect IPW and HA‐MSM to perform particularly poorly if the extreme weights are not truncated. Nevertheless, we still perform the analysis here both with and without truncation to compare the effect of truncating weights.

For these analyses, we must also consider the four assumptions highlighted in Section 3.1: no interference, positivity, consistency, and no unmeasured confounding. Interference should not be an issue, because CF is a non‐infectious condition. Furthermore, people with CF are generally kept out of direct contact with one another to avoid cross‐infection of respiratory microorganisms.^4^ The assumption of positivity was also considered to be valid for this investigation. Although guidelines do exist to help advise when patients might benefit from DNase, it is not uncommon for patients to receive or not receive treatment despite the guidelines. Once DNase treatment has been initiated, it is usual to continue to receive the treatment indefinitely, but a number of people do also stop taking treatment for various reasons. Furthermore, in the IPW analysis, there were no extreme weights, suggesting that the assumption of positivity held. Consistency concerns the definition of the intervention. The standard dosage and frequency of DNase is 2.5 mg once a day, but a small number of patients receive a different dosage or frequency. Unfortunately, dosage data are not routinely collected in the Registry. However, consistency is considered to hold under an intervention defined as “receives DNase as prescribed by doctor'.

All models included the time‐varying covariates ppFEV_1_ and IV days as both outcomes and confounders. The analyses also adjusted for baseline confounders: age, sex, ethnicity, and genotype class (a binary marker of the severity of the CF‐causing mutation). It is possible that there is residual confounding of the treatment‐outcome association and there were a number of other covariates measured in the UK CF Registry that could have been adjusted for, eg, smoking status or body mass index (BMI). However, there is a large amount of missing data in these variables, resulting in many observations being dropped from the analyses if they were included. In sensitivity analyses based on the subset without missing data adjusting for time‐varying smoking status and BMI had only a very small impact on the effect estimates.

Our analysis included 22 357 annual assessments from 3847 people. The median number of visits per person was 8 (IQR, 5‐9). DNase was used for at least 1 year by 2251 people (58.5%) and for at least 5 years by 823 people (21.4%). Table 1 gives an overview of the people included in the analysis at baseline.

**Table 1:** Descriptive baseline statistics of people included in data analysis. Mean (SD) are given for continuous variables and n (%) for categorical variables

Variable	Received DNase During Follow‐Up?	
	No (n = 1596)	Yes (n = 2251)
Age, y	20.8 (13.9)	16.0 (11.7)
ppFEV_1_	84.5 (19.9)	78.6 (20.5)
Annual IV days	5.8 (14.4)	10.6 (19.4)
Sex		
Female	716 (44.9)	1081 (48.0)
Male	880 (55.1)	1170 (52.0)
Ethnicity		
Caucasian	1547 (96.9)	2172 (96.5)
Other	49 (3.1)	79 (3.5)
Genotype class		
High	909 (57.0)	1708 (75.9)
Low	310 (19.4)	183 (8.1)
Unassigned	377 (23.6)	360 (16.0)

### 5.1 Results of lung function analysis

**Figure 10:**
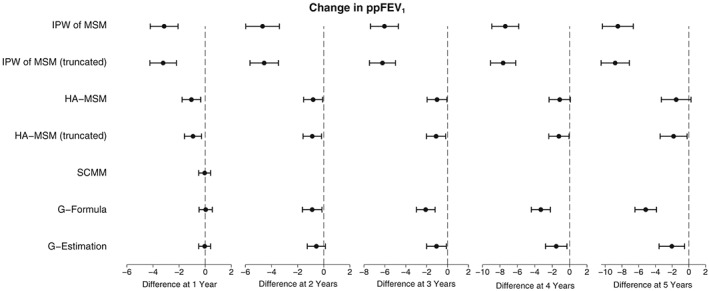
Plots showing the estimated population‐average effect of DNase treatment on ppFEV_1_. HA‐MSM, history‐adjusted marginal structural models; IPW, inverse probability weighting; MSM, marginal structural models; SCMM, sequential conditional mean models

Figure 10 presents the results of the estimated population‐average effect of DNase on ppFEV_1_ depending on length of treatment use. At 1 year, all methods except g‐computation formula estimate that treatment has a negative effect on ppFEV_1_. The results for SCMM, g‐computation formula, and g‐estimation are, however, not significant (*P* = .86, .89, and .86, respectively), whereas IPW and HA‐MSM estimate a stronger, significant, negative effect (*P* < .001 and .005), which does not change much upon truncation of the extreme weights. Looking at longer term effects, all methods showed a trend with the treatment effect becoming more negative through time, with truncated IPW estimating the largest difference in ppFEV_1_ between those taking and not taking treatment of −8.81% (95% CI, −10.50 to −7.12, *P* < .001) and HA‐MSM the smallest effect of −1.52% (95% CI, −3.30 to 0.27, *P* = .097). Full results from this analysis can be found in Table S7.

**Figure 11:**
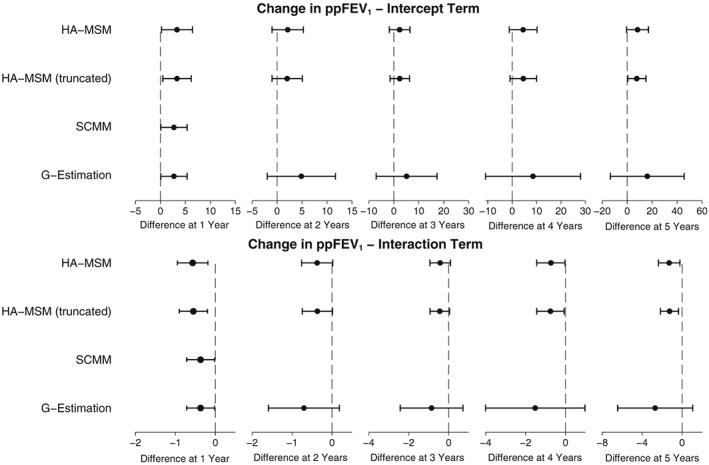
Plots showing the estimated effect of DNase treatment on ppFEV_1_ with effect modification by previous measure of ppFEV_1_. The intercept term is the estimated effect for an individual with ppFEV_1_ equal to 0 in the previous year, and the interaction effect is the estimated change per 10 increase in the previous year's ppFEV_1_. HA‐MSM, history‐adjusted marginal structural models; SCMM, sequential conditional mean models

SCMM, and g‐estimation were also used to investigate effect modification of the treatment effect by time‐varying ppFEV_1_. These results are presented in Figure 11 and show that treatment was estimated to be beneficial in people with lower baselined ppFEV_1_. HA‐MSM estimated an intercept term of 3.32, with treatment becoming less beneficial by 0.57 per 10% change in baseline ppFEV_1_. This equates to a beneficial effect for people with a baseline ppFEV_1_ below 58% and a negative effect for people with ppFEV_1_ above 58%. SCMM and g‐estimation estimated a more attenuated interaction effect where treatment became less effective by 0.37 per 10% change in baseline ppFEV_1_. This means that for these methods, treatment was estimated to be beneficial for people with a baseline ppFEV_1_ up to 73%.

Looking at the 5‐year treatment effect, the interaction between treatment and ppFEV_1_ was estimated to increase in strength leading to a bigger differentiation in effect between those with low and high baseline ppFEV_1_. In the case of HA‐MSM, the intercept was estimated to be 8.30 with a change in effect of −1.29 per 10% increase in ppFEV_1_, leading to a boundary for a beneficial effect of 64%. G‐estimation showed a stronger interaction effect at 5 years of −2.69%, but due to the increased SE, this was not significant (*P* = .16). The full results from the analysis including the interaction term can be seen in Table S8.

### 5.2 Results of IV days analysis

**Figure 12:**
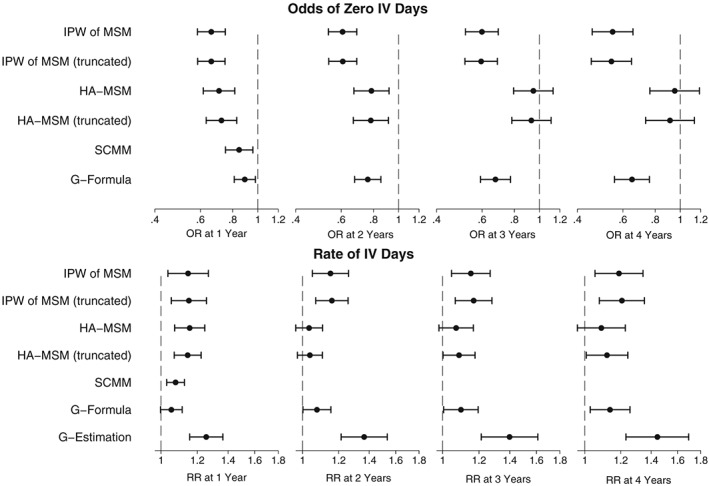
Plots showing the estimated population‐average effect of DNase treatment on annual IV days. HA‐MSM, history‐adjusted marginal structural models; IPW, inverse probability weighting; MSM, marginal structural models; SCMM, sequential conditional mean models

Similarly to the ppFEV_1_ analysis, we generally estimated a negative effect when considering the population average effect of DNase on the annual number of IV days. These results are shown in Figure 12 and can also be seen in more detail in Table S9.

At 1 year, all methods estimated a strong, significant decrease in the odds of having zero IV days and an increase in the overall rate of the number of IV days for those receiving treatment. As we observed in the simulation studies, the estimates from g‐estimation were larger due to the fact that it does not estimate the odds of a zero count separately to the overall rate.

The estimates for the 4‐year treatment effects were very similar to the 1‐year treatment effect estimates, so although treatment was still not estimated to be beneficial, we did not observe a trend of divergence between the treated and nontreated as was observed with ppFEV_1_.

**Figure 13:**
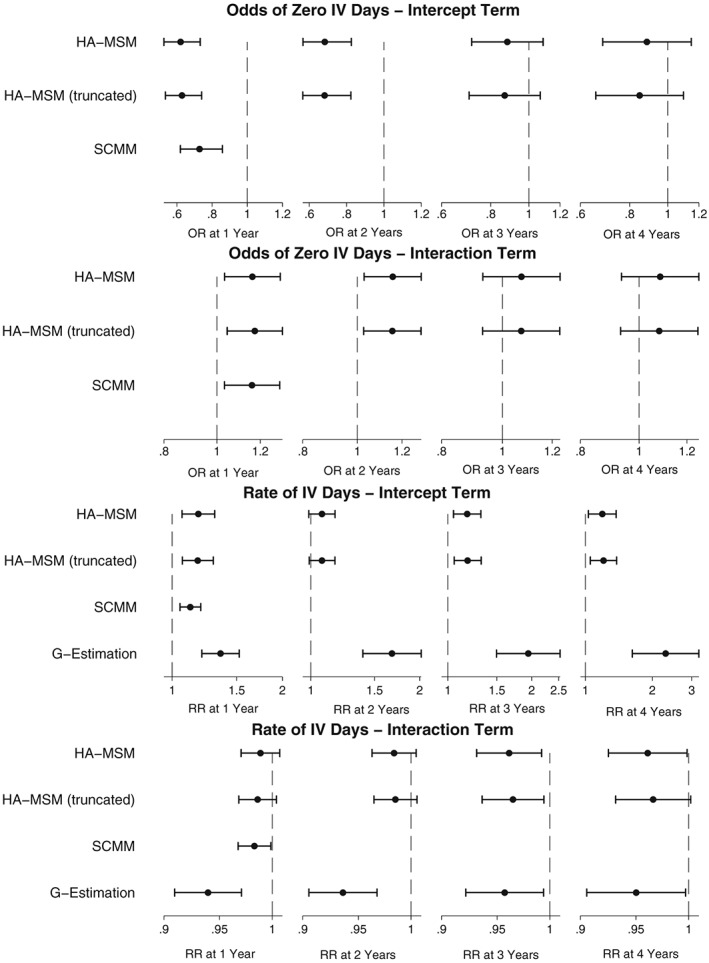
Plots showing the estimated effect of DNase treatment on IV days with effect modification by previous number of IV days. The intercept term is the estimated effect for an individual with 0 IV days in the previous year, and the interaction effect is the estimated change per 10 increase in the number of IV days in the previous year. HA‐MSM, history‐adjusted marginal structural models; SCMM, sequential conditional mean models

The results including an interaction term between previous number of IV days and treatment are shown in Figure 13. In people who had previously had zero IV days, treatment was estimated to decrease their odds of zero future IV days by between 0.62 (HA‐MSM) and 0.73 (SCMM), but for every 10 additional previous IV days, the odds of zero future IV days increased by between 1.16 (HA‐MSM) and 1.17 (truncated HA‐MSM). This means that treatment would be estimated to become beneficial on the odds of zero future IV days in individuals who previously had more than between 21 IV days (SCMM) or 32 IV days (HA‐MSM).

Considering the overall rate of IV days, the interaction effect was not significant for HA‐MSM but was for SCMM and g‐estimation, where for people with zero previous IV days, treatment was estimated to increase the rate of future IV days by between 1.12 (SCMM) and 1.36 (g‐estimation), and this was estimated to decrease by a rate of 0.98 (SCMM) and 0.94 (g‐estimation) per 10 IV days, resulting in a treatment estimated to be beneficial for people with more than 56 previous IV days for SCMM or more than 50 previous IV days (g‐estimation).

By 4 years, the interaction present at 1 year modifying the effect of the odds of a zero count had attenuated from 1.16 to 1.08 and was no longer significant. However, there was moderate evidence of interaction when considering the overall rate of IV days with treatment estimated to be beneficial at 4 years in those who had previously had more than 43 days (HA‐MSM) or 162 IV days (g‐estimation). Table S10 contains the full results from this analysis.

## 6 DISCUSSION

We have investigated the suitability of five methods for estimating treatment effects in longitudinal observational data using simulation studies and applied the methods to the UK CF Registry. The focus was on five features encountered in these investigations (Section 2.2). The suitability and performance of the methods differs depending on the research question, the nature of the treatment effect of interest, and the features of the data. Here, we provide an overview and recommendations based on our findings.

Our simulation studies showed that all the methods we considered are suitable for analysing registry data to investigate treatment effects in many scenarios. Specifically in the standard scenario, where all models are correctly specified, all methods performed very similarly with little impact depending on the method chosen.

In the case of IPW, however, there were noticeable differences between the method with truncated and untruncated weights. In most situations, the untruncated weights performed best, but in the situation of causal pathways being misspecified, the truncated weights showed much better performance. In a real scenario, we would not know which scenario we are in; it would therefore be difficult to know when weights should be truncated or not. It may be sensible to only truncate when there are “extreme” weights, but there is no clear definition of how large a weight must be before it is “extreme.” This would suggest, therefore, in situations where there is uncertainty in the correct direction of causal pathways, that IPW not be used.

HA‐MSM performed similarly to IPW of MSM in cases where there is no effect modification, but as it is a more complex method, it would be preferable to use standard IPW of MSM over HA‐MSM in most cases.

For measuring the 1‐year effect of treatment, SCMM would probably be the preferred method due to its good performance in the simulation studies and its simplicity to implement. The obvious drawback is that the method cannot be used to estimate long‐term effects like the other methods, but we recommend that this method be used alongside other methods to check whether the more complex methods are in agreement with the 1‐year effect estimate of the SCMM. In cases where the 1‐year effect estimate is markedly different between SCMM and another method, this could act as a flag of potential issues with the analysis.

Another benefit of SCMM is that the model‐based standard errors will be approximately correct when the propensity score is well estimated, meaning that the bootstrap does not need to be used and results can be obtained much faster than using the other methods presented in this paper. The asymptotic SEs have been derived for IPW of MSM, but only in a time‐fixed setting,^26^ and the difficulty of deriving these in a longitudinal setting necessitate the use of the bootstrap for all the methods other than SCMM.

G‐computation formula tended to perform as well as other methods, only performing poorly where other methods also performed poorly. The SE were consistently smaller than for other methods, which would always be preferable in cases where we are confident in the specified models for the time‐varying covariates. However, in cases where there is misspecification, the SE remains small, and with real data, it is unlikely that all the assumptions necessary for g‐computation formula would be completely correct, which could result in tight confidence intervals around an incorrect effect estimate. In our scenarios, we did not encounter any issue with the g‐null paradox. This is because, for the g‐null paradox to arise it is necessary for treatment to affect a time‐dependent confounder without having any direct or indirect effect on the outcome.^27^ In our “no effect scenario,” treatment had no effect on either lung function or IV days, which are acting as both the outcome and the time‐dependent confounders.

For continuous outcomes, g‐estimation performed well with the SE generally lying between those of g‐computation formula and IPW, with the advantage that the method can also estimate effect modification by time‐varying covariates, without the drawbacks of unstable weights which were sometimes observed in HA‐MSM. However, with the count outcome, g‐estimation used a gamma model rather than the zero‐inflated negative binomial model like the other methods presented in this paper. This resulted in only one rate ratio compared with the two distinct effect estimates of the other methods making comparison difficult. In situations where the count outcome is not as skewed as the annual IV days in the UK CF registry data, g‐estimation may be a suitable method, but in our setting, the other methods were generally preferable.

We outlined how all methods can handle a count outcome, with the outcome model being restricted to a gamma model in g‐estimation. A further complexity of the count outcome is the issue of non‐collapsibility. In the simulations, we found that when there is truly no treatment effect, both marginal and conditional estimates were correctly consistent with there being no treatment effect. However, in cases where there is a treatment effect, comparison between marginal and conditional estimates from different methods is not as useful.

In addition to the five methods considered in this paper, there are other methods that could have been considered for estimation of treatment effects in the analysis of the Registry data. One such method is targeted maximum likelihood estimation, which is related to the g‐computation formula.^28^ This method has previously been compared with both IPW and the g‐computation formula.^29^, ^30^, ^31^

Considering the analysis of the UK CF Registry data, as hypothesised, there did appear to be effect modification of the treatment effect by previous ppFEV_1_ and previous annual IV days. This resulted in the population average estimates hiding the fact that treatment could be beneficial for a group of people. Therefore, in this situation, we would prefer to use SCMM, HA‐MSM, or g‐estimation, which can estimate effect modification of the treatment effect by time‐varying covariates. Due to the fact that we are unsure of the correct specification of some of the causal pathways, HA‐MSM may not be a suitable method as shown in the simulation studies. However, the results from all four methods (Figure 11) were very similar, suggesting that the direction of the causal pathways may not be misspecified and any of the methods may in fact be suitable.

There was also evidence of effect modification of the treatment effect on the annual number of IV days. However, depending on the method used, treatment was not estimated to become beneficial until individuals had had over at least 21 IV days in the previous year. In our data, almost 80% of people had fewer than 21 IV days, meaning treatment would only be beneficial in reducing IV days in a small subset of people if these results are reliable. However, a further issue with the annual IV days is that people are not only prescribed IVs as a result of an exacerbation of symptoms, but sometimes they are prescribed as a protective measure to avoid a future exacerbation. It is plausible that people who are more likely to be prescribed treatment are also more likely to be prescribed IVs and it may not be possible to account for this confounding with the available data in the Registry. The issue of unmeasured confounding has not been considered in this paper, because it is an assumption of all the considered methods that there is no unmeasured confounding, but it is important to remember this when considering if the data available are suitable for the desired analysis.

Previous work using more traditional statistical methods has only investigated the effects of up to 2 years of DNase treatment. We have shown in this paper how the data available in registries can be harnessed with appropriate statistical methods to investigate the effects of longer term use of treatments. Many treatments for CF would actually be used for more than 5 years, which was the maximum time‐frame considered in this paper due to the limited sample size with follow‐up longer than 5 years, but as more data are collected in the UK CF Registry, further analyses with longer follow‐up could be performed.

Unfortunately, as with a lot of observational data, there are high levels of missingness in some of the variables collected in the UK CF Registry. As missing data were not the focus of this paper, we presented the results of the data analysis with adjustment for variables that are considered to be the strongest confounders affecting the probability of receiving treatment and outcomes, as these variables are also more widely collected. Furthermore, sensitivity analyses suggested that including other potential confounders such as smoking status or BMI did not result in significant changes to the results.

In conclusion, in most settings, more than one of the available methods would be suitable for the types of analysis considered in this paper. In many cases, therefore, it may be beneficial to consider using more than one available method, to see if the results are consistent. Of course, in cases where 2 separate methods give the same effect estimate, this does not mean it is correct, but does add some reliability to the results. In cases where the methods gave very different effect estimates, this would act as a flag to re‐examine the data, the assumptions of the methods, and the suitability of the analyses performed.

## Supporting information

NewsomeSJ_Appendix.pdf
