# Supplementary material for: Estimating long‐term treatment effects in observational data: A comparison of the performance of different methods under real‐world uncertainty
Source: Stat Med. 2018 Apr 19;37(15):2367–90. doi: 10.1002/sim.7664 (PMC6001810; doi:10.1002/sim.7664)
Supplement: Supplementary file 1 — NewsomeSJ_Appendix.pdf [file SIM-37-2367-s001.pdf]

APPENDIX A: SUPPLEMENTARY MATERIAL

A.1 Details of Simulation Studies

We simulated data under six different scenarios. For each scenario 1000 datasets were simulated resulting in moderately independent simulations where for each scenario a new set of datasets was generated, but within each scenario the same set of simulated independent datasets was used to compare the statistical methods.[25]

For each scenario the starting seeds used to generate each simulated dataset were pseudo-random numbers generated from a uniform distribution using the following formula:

Seed = floor(unif(0, 1) $*$ 1000000 + 1000000 $*$ $(n - 1)$) $(1 \leq n \leq 1000)$.

For each scenario datasets were simulated with 7500 individuals with 6 visits ($t = 0, ..., 5$). The data were simulated sequentially starting with visit 0. Table A1 lists the variables that were simulated.

Name	Variable	Type	Distribution	Range
Visit	t	Integer	-	0-5
Censored	c	Binary	Bernoulli	0, 1
Baseline Age	a	Continuous	Beta	6-90
Dornase Alfa	x	Binary	Bernoulli	0, 1
Lung Function	f	Continuous	Normal	10-
IV Days	v	Integer	Zero Inflated Negative Binomial	0-365
Exacerbation (IV Days > 0)	e	Binary	Bernoulli	0, 1

TABLE A1 List of simulated variables

The data for visit 0 were simulated using the following formulae:

$$
\begin{aligned}
a &= 6 + 84 * \text{beta}(1.1, 5) \\
v_0 &= \min\Big(365, \Big(\mathbb{1}\big(\text{unif}(0,1) < \text{expit}(0.004a - 0.6)\big)\Big) * \text{poisson}\Big(\text{gamma}\big(2, 0.5\exp(2.4 - 0.002a)\big)\Big)\Big) \\
e_0 &= \mathbb{1}(v_0 > 0) \\
x_0 &= 0 \\
f_0 &= \max\big(10, \text{norm}(95 - 0.65a - 5.8e_0 - 0.3v_0, 20)\big) \\
c_0 &= 0
\end{aligned}
$$

Data for visits 1 to 5 were then simulated sequentially with the following formulae, where α, β and γ were varied to create the six different scenarios (see Table A2):

$$
\begin{aligned}
v_t = \min\Big(365, \Big(\mathbb{1}\big(\text{unif}(0,1) < \text{expit}(1 + (\alpha_1 + \alpha_2 e_{t-1} + \alpha_3 v_{t-1})x_{t-1} + (\alpha_4 + \alpha_5 e_{t-1} + \alpha_6 v_{t-1}) \sum_{i=0}^{t-1} x_i \\
+ e_{t-1} + 0.05 v_{t-1} - 0.025 f_{t-1} - 0.02a)\big)\Big) \\
* \text{poisson}\Big(\text{gamma}\big(\frac{1}{0.3}, 0.3\exp(3.6 + (\alpha_7 + \alpha_8 e_{t-1} + \alpha_9 v_{t-1})x_{t-1} + (\alpha_1 0 + \alpha_1 1 e_{t-1} + \alpha_1 2 v_{t-1}) \sum_{i=0}^{t-1} x_i \\
+ 0.1 e_{t-1} + 0.01 v_{t-1} - 0.0075 f_{t-1} - 0.003a)\big)\Big)\Big)
\end{aligned}
$$

$$e_t = \mathbb{1}(v_t > 0)$$

$$x_t = \mathbb{1}\big(\text{unif}(0,1) < \text{expit}(4x_{t-1} + 0.7e_t + 0.001v_t - 0.01f_{t-1} - 0.02a - 0.4)\big)$$

$$f_t = \max\big(10, \text{norm}(10 + (\beta_1 + \beta_2 f_{t-1})x_t + (\beta_3 + \beta_4 f_{t-1}) \sum_{i=0}^{t} x_i + 0.9f_{t-1} - 0.7e_t - 0.06v_t - 0.08a, 10)\big)$$

$$c_t = \gamma * \mathbb{1}\big(\text{unif}(0,1) < \text{expit}(0.02a - 0.03f_t - 0.9e_t + 0.02v_t - 0.1x_t - 2\big)$$

The only scenario where the data were simulated differently was the reversed causal pathways scenario, where treatment (x_t) was simulated prior to IV days (v_t), so that in this scenario treatment at visit t depended on the number of IV days at visit $t-1$, and the IV days at visit t depended on treatment at visit t.

**Scenario**	α_1	α_2	α_3	α_4	α_5	α_6	α_7	α_8	α_9	α_{10}	α_{11}	α_{12}	β_1	β_2	β_3	β_4	γ
Standard	-2	0	0	0	0	0	-0.8	0	0	0	0	0	4	0	0	0	0
No Effect	0	0	0	0	0	0	0	0	0	0	0	0	0	0	0	0	0
Decreasing Effect	-2	0	0	0.25	0	0	-0.8	0	0	0.1	0	0	4	0	-0.5	0	0
Effect Modification	-0.8	-0.4	-0.02	0.1	0.05	0.0025	-0.2	-0.2	-0.02	0.025	0.025	0.0025	8	-0.08	-1	0.01	0
Reversed Causal Pathway	-2	0	0	0	0	0	-0.8	0	0	0	0	0	4	0	0	0	0
Censoring	-2	0	0	0	0	0	-0.8	0	0	0	0	0	4	0	0	0	1

TABLE A2 List of simulated variables

The simulations were checked to ensure that the previous formulae resulted in data which is distributed similarly to that observed in the real UK CF registry.

A.2 Stata Code

The following subsections provide the Stata code used to create the simulated data sets and to analyse them.

A.2.1 Code to Simulate Data

The code below can be used to simulate data for all the scenarios described in the paper by changing the numbers in the 'set-up' values section.

```
set type double
set seed 348871

*Obtain random seeds to start each simulated dataset
forvalues i = 1/1000 {
        local S`i' = floor(runiform() * 1000000+1000000*(`i'-1))
}

local sim_number=1
```

```
*****************************************************************************
***********
***********        Set Up Values
***********
*****************************************************************************

*Change values here to change simulation scenario

*Strength of Effect
        *0 = none
        *1 = strong
        local strength=0

*Effect-Modification by Time-Varying Covariates
        *0 = none
        *1 = linear
        local inter=0

*Long-Term Treatment Effect
        *0 = Treatment Effect does not change over time
        *1 = Treatment effect decreases over time
        local long=0

*Direction of Causal Pathway
        *1 = iv -> da
        *2 = da -> iv
        local path=1

*Censoring
        *0=No Censoring
        *1=Censoring
        local cens=0

*****************************************************************************
***********
***********        Scenario Settings
***********
*****************************************************************************

*Code below sets up treatment effects depending on the scenario given above
if ‘strength’==0 {
        local a1=0 //Exac
        local b1=0 //Exac Int Exac
        local b2=0 //Exac Int IV
        local c1=0
        local c2=0
        local c3=0
        local a2=0 //IV
        local b3=0 //IV Int Exac
        local b4=0 //IV Int IV
        local c4=0
```

```
        local c5=0
        local c6=0
        local a3=0 //FEV
        local b5=0 //FEV Int
        local c7=0
        local c8=0
}
else if ‘strength’==1 & ‘inter’==0 & ‘long’==0 {
        local a1=2 //Exac
        local b1=0 //Exac Int Exac
        local b2=0 //Exac Int IV
        local c1=0
        local c2=0
        local c3=0
        local a2=0.8 //IV
        local b3=0 //IV Int Exac
        local b4=0 //IV Int IV
        local c4=0
        local c5=0
        local c6=0
        local a3=4 //FEV
        local b5=0 //FEV Int
        local c7=0
        local c8=0
}
else if ‘strength’==1 & ‘inter’==0 & ‘long’==1 {
        local a1=2 //Exac
        local b1=0 //Exac Int Exac
        local b2=0 //Exac Int IV
        local c1=0.25
        local c2=0
        local c3=0
        local a2=0.8 //IV
        local b3=0 //IV Int Exac
        local b4=0 //IV Int IV
        local c4=0.1
        local c5=0
        local c6=0
        local a3=4 //FEV
        local b5=0 //FEV Int
        local c7=0.5
        local c8=0
}
else if ‘strength’==1 & ‘inter’==1 & ‘long’==0 {
        local a1=0.8 //Exac
        local b1=0.4 //Exac Int Exac
        local b2=0.02 //Exac Int IV
        local c1=0
        local c2=0
        local c3=0
        local a2=0.2 //IV
```

```
        local b3=0.2 //IV Int Exac
        local b4=0.02 //IV Int IV
        local c4=0
        local c5=0
        local c6=0
        local a3=8 //FEV
        local b5=0.08 //FEV Int
        local c7=0
        local c8=0
}
else if ‘strength’==1 & ‘inter’==1 & ‘long’==1 {
        local a1=0.8 //Exac
        local b1=0.4 //Exac Int Exac
        local b2=0.02 //Exac Int IV
        local c1=0.1
        local c2=0.05
        local c3=0.0025
        local a2=0.2 //IV
        local b3=0.2 //IV Int Exac
        local b4=0.02 //IV Int IV
        local c4=0.025
        local c5=0.025
        local c6=0.0025
        local a3=8 //FEV
        local b5=0.8 //FEV Int
        local c7=1
        local c8=0.01
}

*****************************************************************************
***********
***********        Simulate Data
***********
*****************************************************************************

qui forv m=1/1000 {
        set seed ‘S‘sim_number’’

        *7500 individuals with 6 visits each
        set obs 45000
        gen id=ceil(_n/6)
        bysort id: gen visit=_n

        ***************
        * FIRST VISIT *
        if ‘cens’==0 {
                gen year=2007 if visit==1
        }
        else {
                gen temp=uniform()
                gen year=cond(temp<0.4,2007,cond(temp<0.6,2008,cond(temp<0.75,2009, ///
```

```
                cond(temp<0.85,2010,cond(temp<0.95,2011,2012)))))
drop temp
}

gen Bage=6+84*rbeta(1.1,5) if visit==1 & year==2007
replace Bage=6+84*rbeta(0.65,3.5) if visit==1 & year!=2007

gen censor=0 if visit==1

gen iv_days=min(365,(uniform()<invlogit(-0.6+0.004*Bage))* ///
        rpoisson(rgamma(2,0.5*exp(3.4-0.002*Bage)))) if visit==1
gen exac=0 if iv_days==0
replace exac=1 if iv_days>0 & iv_days!=.

gen dnase=0 if visit==1
gen cum_dnase=dnase

gen fev1=max(10,rnormal(95-0.65*Bage-5.8*exac-0.3*iv_days,20)) if visit==1

*********************
* Subsequent Visits *

*Use Age At First Visit for all Visits*
by id: replace Bage=Bage[_n-1] if _n!=1

*Simulate Dornase Alfa & FEV1 Sequentially through time *
qui forv n=2/6 {

        replace year=year[_n-1]+1 if visit==`n'

        if `path'==1 {
                replace iv_days=(uniform()<invlogit(1-`a1'*dnase[_n-1] ///
                        -`b1'*dnase[_n-1]*exac[_n-1] -`b2'*dnase[_n-1]*iv_days[_n-1] ///
                        +`c1'*cum_dnase[_n-1] ///
                        +`c2'*cum_dnase[_n-1]*exac[_n-1] ///
                        +`c3'*cum_dnase[_n-1]*iv_days[_n-1] ///
                        -0.025*fev1[_n-1]+exac[_n-1]+0.05*iv_days[_n-1]-0.02*Bage))* ///
                        rpoisson(rgamma(1/0.3,0.3*exp(3.6-`a2'*dnase[_n-1] ///
                        -`b3'*dnase[_n-1]*exac[_n-1]-`b4'*dnase[_n-1]*iv_days[_n-1] ///
                        +`c4'*cum_dnase[_n-1] ///
                        +`c5'*cum_dnase[_n-1]*exac[_n-1] ///
                        +`c6'*cum_dnase[_n-1]*iv_days[_n-1] ///
                        -0.0075*fev1[_n-1]+0.1*exac[_n-1]+0.01*iv_days[_n-1] ///
                        -0.003*Bage))) if visit==`n'

                replace exac=0 if iv_days==0 & visit==`n'
                replace exac=1 if iv_days>0 & iv_days!=. & visit==`n'

                replace dnase=uniform()<invlogit(-0.4+4*dnase[_n-1] ///
                        -0.01*fev1[_n-1]+0.7*exac+0.001*iv_days-0.02*Bage) if visit==`n'
                replace cum_dnase=dnase+cum_dnase[_n-1] if visit==`n'
```

```
        }
        else if ‘path’==2 {
                replace dnase=uniform()<invlogit(-0.4+4*dnase[_n-1] ///
                        -0.01*fev1[_n-1]+0.7*exac[_n-1]+0.001*iv_days[_n-1] ///
                        -0.02*Bage) if visit==‘n’
                replace cum_dnase=dnase+cum_dnase[_n-1] if visit==‘n’

                replace iv_days=(uniform()<invlogit(1-‘a1’*dnase ///
                        -‘b1’*dnase*exac[_n-1] -‘b2’*dnase*iv_days[_n-1] ///
                        +‘c1’*cum_dnase ///
                        +‘c2’*cum_dnase*exac[_n-1] ///
                        +‘c3’*cum_dnase*iv_days[_n-1] ///
                        -0.025*fev1[_n-1]+exac[_n-1]+0.05*iv_days[_n-1]-0.02*Bage))* ///
                        rpoisson(rgamma(1/0.3,0.3*exp(3.6-‘a2’*dnase ///
                        -‘b3’*dnase*exac[_n-1]-‘b4’*dnase*iv_days[_n-1] ///
                        +‘c4’*cum_dnase ///
                        +‘c5’*cum_dnase*exac[_n-1] ///
                        +‘c6’*cum_dnase*iv_days[_n-1] ///
                        -0.0075*fev1[_n-1]+0.1*exac[_n-1]+0.01*iv_days[_n-1] ///
                        -0.003*Bage))) if visit==‘n’

                replace exac=0 if iv_days==0 & visit==‘n’
                replace exac=1 if iv_days>0 & iv_days!=. & visit==‘n’
        }

        replace fev1=rnormal(10+‘a3’*dnase-‘b5’*dnase*fev1[_n-1] ///
                -‘c7’*cum_dnase + ‘c8’*cum_dnase*fev1[_n-1] ///
                +0.9*fev1[_n-1]-0.7*exac-0.06*iv_days-0.08*Bage,10) if visit==‘n’

        if ‘cens’==0 {
                replace iv_days=365 if iv_days>365 & visit==‘n’
                replace fev1=10 if fev1<10 & visit==‘n’
                replace censor=0 if visit==‘n’
        }
        else if ‘cens’==1 {
                replace censor=year>2012|fev1<10|iv_days>365 | ///
                        uniform()<invlogit(0.02*Bage-0.03*fev1-0.9*exac+0.02*iv_days ///
                        -0.1*dnase-2) if visit==‘n’
                replace iv_days=. if visit==‘n’ & censor==1
                replace exac=. if visit==‘n’ & censor==1
                replace dnase=. if visit==‘n’ & censor==1
                replace cum_dnase=. if visit==‘n’ & censor==1
                replace fev1=. if visit==‘n’ & censor==1
        }
}

*Make Lagged Variables
qui makelag dnase fev1 cum_dnase exac iv_days, firstvis(1) visit(visit)
qui makelag dnase fev1 cum_dnase exac iv_days Ldnase Lcum_dnase, firstvis(1) visit(visit)

drop if censor==1
```

```
        drop censor
        gen censor=0
        sort id year
        bysort id: replace censor=1 if year!=2012 & _n==_N

        *Save Simulated Dataset
        save "Scenario1/sim`m'", replace
        clear
        local sim_number=`sim_number'+1
}
```

A.2.2 Code to Analyse Data

The following code was used to analyse the simulated datasets.

```
************* Models 1 & 2 - SCMM - With/Without Interactions
*************        Lung Funcation & IV Days
*******************************************************

*Propensity Score
qui logit dnase i.Ldnase##c.LLfev1 i.Ldnase##i.exac i.Ldnase##c.iv_days Bage
qui predict ps, pr
sort id visit
by id: gen ps2=ps[_n-1] if _n!=1

*Final Model - Without Interactions - Lung Function
regress fev1 i.dnase Lfev1 c.Lcum_dnase Bage i.exac iv_days ps, cluster(id)

*Final Model - Without Interactions - IV Days
zinb iv_days i.Ldnase i.Lexac Liv_days LLcum_dnase LLfev1 Bage ps2, ///
        inflate(i.Ldnase i.Lexac Liv_days LLcum_dnase LLfev1 Bage ps2) cluster(id)

*Final Model - With Interactions - Lung Function
regress fev1 i.dnase##c.Lfev1 c.Lcum_dnase##c.Lfev1 Bage i.exac iv_days ///
        c.ps##c.Lfev1, cluster(id)

*Final Model - With Interactions - IV Days
zinb iv_days i.Ldnase##i.Lexac i.Ldnase##c.Liv_days c.LLcum_dnase##i.Lexac ///
        c.LLcum_dnase##c.Liv_days LLfev1 Bage c.ps2##i.Lexac c.ps2##c.Liv_days, ///
        inflate(i.Ldnase##i.Lexac i.Ldnase##c.Liv_days c.LLcum_dnase##i.Lexac ///
        c.LLcum_dnase##c.Liv_days LLfev1 Bage c.ps2##i.Lexac c.ps2##c.Liv_days) cluster(id)

************* Model 3 & 4 - IPW - Truncated/Non-Truncated Weights
*************        Lung Function
*******************************************************

*Denominator*
qui logit dnase i.Ldnase##c.Lfev1 i.Ldnase##i.exac i.Ldnase##c.iv_days Bage
qui predict denom, pr
qui replace denom=1-denom if dnase==0

*Numerator*
```

```
qui logit dnase i.Ldnase Bage
qui predict num, pr
qui replace num=1-num if dnase==0

*Censoring Denominator*
capture noisily logit censor i.dnase fev1 i.exac iv_days Bage if visit!=6, asis
        if _rc==0 {
        predict denom_cen if e(sample), pr
        replace denom_cen=1-denom_cen
        replace denom_cen=1 if visit==6
}
else {
        gen denom_cen=1
}

*Censoring Numerator*
capture noisily logit censor Bage if visit!=6, asis
if _rc==0 {
        predict num_cen if e(sample), pr
        replace num_cen=1-num_cen
        replace num_cen=1 if visit==6
}
else {
        gen num_cen=1
}

qui gen stab=num/denom
qui gen cen=num_cen/denom_cen
sort id visit
by id: gen cen2=cen[_n-1] if _n!=1
by id: replace cen2=1 if _n==1

qui sort id visit
qui by id: replace stab=stab*stab[_n-1] if _n>2
qui by id: replace cen2=cen2*cen2[_n-1] if _n>1
qui replace stab=stab*cen2

*Final Model Without Truncated Weights
regress fev1 i.dnase##i.Lcum_dnase Bage i.visit [pw=stab], cluster(id)

qui sum stab, d
replace stab=r(p99) if stab>r(p99) & stab!=.
replace stab=r(p1) if stab<r(p1)

*Final Model With Truncated Weights
regress fev1 i.dnase##i.Lcum_dnase Bage i.visit [pw=stab], cluster(id)

************* Model 3 & 4 - IPW - Non-Truncated & Truncated Weights
*************        IV Days
*******************************************************
```

```
*Denominator*
qui logit dnase i.Ldnase##c.Lfev1 i.Ldnase##i.exac i.Ldnase##c.iv_days Bage if visit!=6
qui predict denom if e(sample), pr
qui replace denom=1-denom if dnase==0

*Numerator*
qui logit dnase i.Ldnase if visit!=6
qui predict num if e(sample), pr
qui replace num=1-num if dnase==0

*Censoring Denominator*
capture noisily logit censor i.dnase fev1 i.exac iv_days Bage if visit!=6, asis
if _rc==0 {
        predict denom_cen if e(sample), pr
        replace denom_cen=1-denom_cen
        replace denom_cen=1 if visit==6
}
else {
        gen denom_cen=1
}

*Censoring Numerator*
capture noisily logit censor if visit!=6,  asis
if _rc==0 {
        predict num_cen if e(sample), pr
        replace num_cen=1-num_cen
        replace num_cen=1 if visit==6
}
else {
        gen num_cen=1
}

qui gen stab=num/denom
qui gen cen=num_cen/denom_cen

qui sort id visit
qui by id: replace stab=stab*stab[_n-1] if _n>2
qui by id: replace cen=cen*cen[_n-1] if _n>1
qui replace stab=stab*cen

qui by id: gen stab2=stab[_n-1] if _n!=1

*Final Model - Non-Truncated Weights
zinb iv_days i.Ldnase##i.LLcum_dnase i.visit [pw=stab2], ///
        inflate(i.Ldnase##i.LLcum_dnase i.visit) cluster(id)

qui sum stab2, d
replace stab2=r(p99) if stab2>r(p99) & stab2!=.
replace stab2=r(p1) if stab2<r(p1)

*Final Model - Truncated Weights
```

```
zinb iv_days i.Ldnase##i.LLcum_dnase i.visit [pw=stab2], ///
        inflate(i.Ldnase##i.LLcum_dnase i.visit) cluster(id)

****************************************************************
*************       Models 5, 6, 7 & 8 - History-Adjusted MSM
*************       Non-Truncated/Trunctaed & With/Without Interactions
*************       Lung Function
***************************************************************

*Step 1 - Create Weights at each Visit
*Denominator*
qui logit dnase i.Ldnase##c.Lfev1 i.Ldnase##i.exac i.Ldnase##c.iv_days Bage
qui predict denom, pr
qui replace denom=1-denom if dnase==0

*Numerator*
qui logit dnase i.Ldnase Bage, iterate(50) asis
qui predict num, pr
qui replace num=1-num if dnase==0

qui gen stab=num/denom

*CENSORING WEIGHTS
*Censoring Denominator*
capture noisily logit censor i.dnase fev1 i.exac iv_days Bage if visit!=6, asis
if _rc==0 {
        predict denom_cen if e(sample), pr
        replace denom_cen=1-denom_cen
        replace denom_cen=1 if visit==6
}
else {
        gen denom_cen=1
}

*Censoring Numerator*
capture noisily logit censor Bage if visit!=6, asis
if _rc==0 {
        predict num_cen if e(sample), pr
        replace num_cen=1-num_cen
        replace num_cen=1 if visit==6
}
else {
        gen num_cen=1
}

qui gen cen=num_cen/denom_cen
sort id visit
by id: gen cen2=cen[_n-1] if _n!=1
by id: replace cen2=1 if _n==1
replace cen=cen2
drop cen2
```

```
replace stab=stab*cen

*Step 2 - Expand Data
*Create all the rows that are needed for all s & t combinations
rename visit t
gen expand=N-t+1
qui expand expand, gen(duplicate)
drop expand

*Generate s and Sort Data
bysort id t: gen s=N-_n+1
gsort id s -t

gen smt=s-t

*Need FEV1 at Visit S for all t
gen Ys=fev1 if s==t
by id s: replace Ys=Ys[_n-1] if _n!=1

*Step 3 - Multiply Weights
by id s: gen new_stab=stab[_n-1] if _n!=1
replace new_stab=1 if s==t
by id s: replace new_stab=new_stab*new_stab[_n-1] if _n!=1

*Step 3a - Create Truncated Weights
        gen new_stab2=new_stab
        qui sum new_stab2, d
        replace new_stab2=r(p99) if new_stab2>r(p99) & new_stab2!=.
        replace new_stab2=r(p1) if new_stab2<r(p1)


*Step 4 - Fit MSM
*Set-Up Cumulative Dnase Variables
by id s: gen max_dnase=cum_dnase[1]
gen dnase_1=dnase if max_dnase==cum_dnase
replace dnase_1=dnase_1[_n-1] if dnase_1[_n-1]==1 & max_dnase!=cum_dnase
qui forval n=2/5 {
        gen dnase_‘n’=dnase if max_dnase==cum_dnase+‘n’-1
        replace dnase_‘n’=dnase_‘n’[_n-1] if dnase_‘n’[_n-1]==1 & max_dnase>cum_dnase+1
        recode dnase_‘n’ .=0
}

*Final Model - Non-Truncated Weights - No Interaction
regress Ys i.dnase_? i.Lcum_dnase##i.smt c.Lfev1##i.smt c.Bage##i.smt ///
        i.exac##i.smt c.iv_days##i.smt [pw=new_stab], cluster(id)

*Final Model - Non-Truncated Weights - With Interaction
regress Ys i.dnase_?##c.Lfev1 i.Lcum_dnase##i.smt c.Lfev1##i.smt ///
        c.Bage##i.smt i.exac##i.smt c.iv_days##i.smt [pw=new_stab], cluster(id)
```

```
*Final Model - Truncated Weights - No Interaction
regress Ys i.dnase_? i.Lcum_dnase##i.smt c.Lfev1##i.smt c.Bage##i.smt ///
        i.exac##i.smt c.iv_days##i.smt [pw=new_stab2], cluster(id)

*Final Model - Truncated Weights - With Interaction
regress Ys i.dnase_?##c.Lfev1 i.Lcum_dnase##i.smt c.Lfev1##i.smt ///
        c.Bage##i.smt i.exac##i.smt c.iv_days##i.smt [pw=new_stab2], cluster(id)

**************************************************************
*************       Models 5, 6, 7, 8 - History-Adjusted MSM
*************       Non-Truncated/Truncated and  With/Without Interactions
*************       IV Days
***************************************************************

*Step 1 - Create Weights at each Visit
*Denominator*
qui logit dnase i.Ldnase##c.Lfev1 i.Ldnase##i.exac i.Ldnase##c.iv_days ///
        Bage if visit!=6
qui predict denom if e(sample), pr
qui replace denom=1-denom if dnase==0

*Numerator*
qui logit dnase i.Ldnase Bage if visit!=6
qui predict num if e(sample), pr
qui replace num=1-num if dnase==0

qui gen stab=num/denom
qui sort id visit
qui by id: gen stab2=stab[_n-1] if _n!=1
replace stab=stab2
drop stab2

*CENSORING WEIGHTS
*Censoring Denominator*
capture noisily logit censor i.dnase fev1 i.exac iv_days Bage if visit!=6, asis
if _rc==0 {
        predict denom_cen if e(sample), pr
        replace denom_cen=1-denom_cen
        replace denom_cen=1 if visit==6
}
else {
        gen denom_cen=1
}

*Censoring Numerator*
capture noisily logit censor Bage if visit!=6, asis
if _rc==0 {
        predict num_cen if e(sample), pr
        replace num_cen=1-num_cen
        replace num_cen=1 if visit==6
}
```

```
else {
        gen num_cen=1
}

qui gen cen=num_cen/denom_cen
sort id visit
by id: gen cen2=cen[_n-1] if _n!=1
by id: replace cen2=1 if _n==1
replace cen=cen2
drop cen2

replace stab=stab*cen

*Step 2 - Expand Data
*Create all the rows that are needed for all s & t combinations
rename visit t
gen expand=N-t+1
qui expand expand, gen(duplicate)
drop expand

*Generate s and Sort Data
bysort id t: gen s=N-_n+1
gsort id s -t

gen smt=s-t

*Need IV Days at Visit S for all t
gen Ys=iv_days if s==t
by id s: replace Ys=Ys[_n-1] if _n!=1

*Step 3 - Multiply Weights
by id s: gen new_stab=stab[_n-1] if _n!=1
replace new_stab=1 if s==t
by id s: replace new_stab=new_stab*new_stab[_n-1] if _n!=1

*Step 3a - Truncate Weights
gen new_stab2=new_stab
qui sum new_stab2, d
replace new_stab2=r(p99) if new_stab2>r(p99) & new_stab2!=.
replace new_stab2=r(p1) if new_stab2<r(p1)

*Step 4 - Fit MSM
by id s: gen max_dnase=Lcum_dnase[1]
gen Ldnase_1=Ldnase if max_dnase==Lcum_dnase
replace Ldnase_1=Ldnase_1[_n-1] if Ldnase_1[_n-1]==1 & max_dnase!=Lcum_dnase
qui forval n=2/4 {
        gen Ldnase_`n'=Ldnase if max_dnase==Lcum_dnase+`n'-1
        replace Ldnase_`n'=Ldnase_`n'[_n-1] if Ldnase_`n'[_n-1]==1 & max_dnase>Lcum_dnase+1
        recode Ldnase_`n' .=0
}
```

```
*Final Model - Non-Truncated Weights, No Interactions
zinb Ys i.Ldnase_? i.Lexac##i.smt c.Liv_days##i.smt i.LLcum_dnase##i.smt ///
        c.LLfev1##i.smt c.Bage##i.smt [pw=new_stab], inflate(i.Ldnase_? ///
        i.Lexac##i.smt c.Liv_days##i.smt i.LLcum_dnase##i.smt c.LLfev1##i.smt ///
        c.Bage##i.smt) cluster(id)

*Final Model - Non-Truncated Weights, With Interactions
zinb Ys i.Ldnase_?##i.Lexac i.Ldnase_?##c.Liv_days i.Lexac##i.smt ///
        c.Liv_days##i.smt i.LLcum_dnase##i.smt c.LLfev1##i.smt c.Bage##i.smt ///
        [pw=new_stab], inflate(i.Ldnase_?##i.Lexac i.Ldnase_?##c.Liv_days ///
        i.Lexac##i.smt c.Liv_days##i.smt i.LLcum_dnase##i.smt c.LLfev1##i.smt ///
        c.Bage##i.smt) cluster(id)

*Final Model - Truncated Weights, No Interactions
zinb Ys i.Ldnase_? i.Lexac##i.smt c.Liv_days##i.smt i.LLcum_dnase##i.smt ///
        c.LLfev1##i.smt c.Bage##i.smt [pw=new_stab2], inflate(i.Ldnase_? ///
        i.Lexac##i.smt c.Liv_days##i.smt i.LLcum_dnase##i.smt c.LLfev1##i.smt ///
        c.Bage##i.smt) cluster(id)

*Final Model - Truncated Weights, With Interactions
zinb Ys i.Ldnase_?##i.Lexac i.Ldnase_?##c.Liv_days i.Lexac##i.smt ///
        c.Liv_days##i.smt i.LLcum_dnase##i.smt c.LLfev1##i.smt c.Bage##i.smt ///
        [pw=new_stab2], inflate(i.Ldnase_?##i.Lexac i.Ldnase_?##c.Liv_days ///
        i.Lexac##i.smt c.Liv_days##i.smt i.LLcum_dnase##i.smt c.LLfev1##i.smt ///
        c.Bage##i.smt) cluster(id)

************* Model 9 - G Formula
*************        Lung Function &        IV Days
***************************************************************

sort id visit
*Generate Space for Simulated Values*
qui forv n=1/6 {
        gen dnase_`n'=0
        replace dnase_`n'=1 if `n'<visit
}
qui forv n=1/6 {
        *Main Variables*
        gen fev1_`n'=fev1 if visit==1
        gen exac_`n'=exac if visit==1|visit==2
        gen iv_days_`n'=iv_days if visit==1|visit==2
        gen cum_dnase_`n'=dnase_`n'
        by id: replace cum_dnase_`n'=cum_dnase_`n'+cum_dnase_`n'[_n-1] if ///
        cum_dnase_`n'!=0 & _n!=1
}

*Conditional Distributions for Confounders - SCMM
regress fev1 i.dnase Lfev1 c.Lcum_dnase        Bage i.exac iv_days
        qui gen lung_Lfev1 = _b[Lfev1]
        qui gen lung_dnase = _b[1.dnase]
        qui gen lung_dnase1 = _b[Lcum_dnase]
```

```
        qui gen lung_Bage = _b[Bage]
        qui gen lung_exac = _b[1.exac]
        qui gen lung_ivdays = _b[iv_days]
        qui gen lung_cons = _b[_cons]
        gen lung_rand=e(rmse)

zinb iv_days i.Ldnase i.Lexac Liv_days c.LLcum_dnase Lfev1 Bage, ///
        inflate(i.Ldnase i.Lexac Liv_days c.LLcum_dnase Lfev1 Bage)
        qui gen iv_dnase = _b[iv_days:1.Ldnase]
        qui gen iv_Lexac = _b[iv_days:1.Lexac]
        qui gen iv_Liv_days = _b[iv_days:Liv_days]
        qui gen iv_dnase1 = _b[iv_days:LLcum_dnase]
        qui gen iv_Bage = _b[iv_days:Bage]
        qui gen iv_Lfev1 = _b[iv_days:Lfev1]
        qui gen iv_cons = _b[iv_days:_cons]
        qui gen exac_dnase = _b[inflate:1.Ldnase]
        qui gen exac_Lexac = _b[inflate:1.Lexac]
        qui gen exac_Liv_days = _b[inflate:Liv_days]
        qui gen exac_dnase1 = _b[inflate:LLcum_dnase]
        qui gen exac_Bage = _b[inflate:Bage]
        qui gen exac_Lfev1 = _b[inflate:Lfev1]
        qui gen exac_cons = _b[inflate:_cons]
        qui gen alpha = exp(_b[lnalpha:_cons])

*Start Simulating Data from Visit 2 for lung function
*Visit 3-6 for all variables
forv m= 1/6 {
        *Update Lung Function
        replace fev1_‘m’=max(10,lung_cons+fev1_‘m’[_n-1]*lung_Lfev1 ///
                +dnase_‘m’*lung_dnase+cum_dnase_‘m’[_n-1]*lung_dnase1 ///
                +Bage*lung_Bage +exac_‘m’*lung_exac+iv_days_‘m’*lung_ivdays ///
                +lung_rand*rnormal()) if visit==2
        }

qui forval n=3/6 {
        *Update Exacerbations*
        *Update IV Days*
        forv m= 1/6 {
                *Update IV Days
                replace iv_days_‘m’=min(365,(runiform()>invlogit(exac_cons ///
                        +fev1_‘m’[_n-1]*exac_Lfev1+dnase_‘m’[_n-1]*exac_dnase ///
                        +exac_‘m’[_n-1]*exac_Lexac+iv_days_‘m’[_n-1]*exac_Liv_days ///
                        +cum_dnase_‘m’[_n-2]*exac_dnase1 +Bage*exac_Bage))* ///
                        rpoisson(rgamma(1/alpha,alpha*exp(iv_cons+fev1_‘m’[_n-1]*iv_Lfev1 ///
                        +dnase_‘m’[_n-1]*iv_dnase+exac_‘m’[_n-1]*iv_Lexac ///
                        +iv_days_‘m’[_n-1]*iv_Liv_days+cum_dnase_‘m’[_n-2]*iv_dnase1 ///
                        +Bage*iv_Bage)))) if visit==‘n’

        replace exac_‘m’=0 if iv_days_‘m’==0
        replace exac_‘m’=1 if iv_days_‘m’>0 & iv_days_‘m’!=.
```

```
        *Update Lung Function
        replace fev1_`m'=max(10,lung_cons+fev1_`m'[_n-1]*lung_Lfev1 ///
                +dnase_`m'*lung_dnase+cum_dnase_`m'[_n-1]*lung_dnase1 ///
                +Bage*lung_Bage +exac_`m'*lung_exac+iv_days_`m'*lung_ivdays ///
                +lung_rand*rnormal()) if visit==`n'
        }
}
sort id visit
forv n=1/6 {
        by id: gen Lcum_dnase_`n'=cum_dnase_`n'[_n-1] if _n!=1
}

drop dnase cum_dnase Bage fev1 iv_days exac Ldnase Lfev1 Lcum_dnase ///
        Lexac Liv_days LLdnase LLcum_dnase lung_Lfev1-lung_rand iv_Lfev1-alpha
gen i=_n
reshape long dnase_ fev1_ cum_dnase_ Lcum_dnase_ exac_ iv_days_, i(i) j(scenario)

*Final Model - Lung Function
regress fev1_ i.cum_dnase i.visit if visit!=1

*Final Model - IV Days
zinb iv_days_ i.Lcum_dnase_ i.visit, inflate(i.Lcum_dnase_ i.visit) cluster(id)

************* Model 10 - G Estimation - Without Interactions
*************        Lung Function
***************************************************************

*Propensity Score
qui logit dnase i.Ldnase##c.Lfev1 i.Ldnase##i.exac i.Ldnase##c.iv_days Bage
qui predict ps, pr

*Censoring Denominator*
capture noisily logit censor i.dnase fev1 i.exac iv_days Bage if visit!=6, asis
if _rc==0 {
        predict denom_cen if e(sample), pr
        replace denom_cen=1-denom_cen
        replace denom_cen=1 if visit==6
}
else {
        gen denom_cen=1
}

*Censoring Numerator*
capture noisily logit censor Bage if visit!=6, asis
if _rc==0 {
        predict num_cen if e(sample), pr
        replace num_cen=1-num_cen
        replace num_cen=1 if visit==6
}
else {
        gen num_cen=1
```

```
}

gen stab=num_cen/denom_cen
sort id visit
by id: gen stab2=stab[_n-1] if _n!=1
recode stab2 .=1
drop stab
rename stab2 stab

*Create all the rows that are needed for all s & t combinations
rename visit t
by id: gen N=_N
gen expand=N-t+1
qui expand expand, gen(duplicate)
drop expand

*Generate s and Sort Data
bysort id t: gen s=N-_n+1
gsort id s -t

*Sort Out Censoring Weights*
by id s: gen stab2=stab[_n-1] if _n!=1
replace stab2=1 if stab2==.
by id s: replace stab2=stab2*stab2[_n-1] if _n!=1

*Need FEV1 at Visit S for all t
gen Ys=fev1 if s==t
by id s: replace Ys=Ys[_n-1] if _n!=1

*Create Z & Z interactions with Dnase & Propensity Score
*Create space to store Phi estimates
qui forv n=1/5 {
        gen z`n'=s>=t+`n'-1
        gen dnase_z`n'=dnase*z`n'
        gen ps_z`n'=ps*z`n'
        gen phi`n'=.
}

*First Iteration Can only Fit Short-Term Model
*i.e. s==t
gen H=Ys if s==t

gen smt=s-t

*Loop through seven times, each time can estimate an addition Phi
qui forv n=1/5 {

        *Get Estimate
        regress H i.dnase_z* ps_z* c.Lcum_dnase##i.smt c.Lfev1##i.smt ///
                c.Bage##i.smt i.exac##i.smt c.iv_days##i.smt if s<t+`n' [pw=stab2]
        forv m=1/5 {
```

```
                capture noisily replace phi`m'=_b[1.dnase_z`m']
        }

        *Create counterfactuals
        replace H=Ys if s==t
        replace H=H[_n-1] - phi1*dnase_z1[_n-1] if s==t+1
        replace H=H[_n-1] - phi1*dnase_z1[_n-1] - phi2*dnase_z2[_n-1] if s==t+2
        replace H=H[_n-1] - phi1*dnase_z1[_n-1] - phi2*dnase_z2[_n-1] ///
                - phi3*dnase_z3[_n-1] if s==t+3
        replace H=H[_n-1] - phi1*dnase_z1[_n-1] - phi2*dnase_z2[_n-1] ///
                - phi3*dnase_z3[_n-1] - phi4*dnase_z4[_n-1] if s==t+4
        replace H=H[_n-1] - phi1*dnase_z1[_n-1] - phi2*dnase_z2[_n-1] ///
                - phi3*dnase_z3[_n-1] - phi4*dnase_z4[_n-1] - phi5*dnase_z5[_n-1] if s==t+5
}

*Final Estimates
regress H i.dnase_z* ps_z* c.Lcum_dnase##i.smt c.Lfev1##i.smt c.Bage##i.smt ///
i.exac##i.smt c.iv_days##i.smt [pw=stab2], cluster(id)

************* Model 11 - G Estimation - With Interactions
*************        Lung Function
****************************************************************

*Propensity Score
qui logit dnase i.Ldnase##c.Lfev1 i.Ldnase##i.exac i.Ldnase##c.iv_days Bage
qui predict ps, pr

*Censoring Denominator*
capture noisily logit censor i.dnase fev1 i.exac iv_days Bage if visit!=6, asis
if _rc==0 {
        predict denom_cen if e(sample), pr
        replace denom_cen=1-denom_cen
        replace denom_cen=1 if visit==6
}
else {
        gen denom_cen=1
}

*Censoring Numerator*
capture noisily logit censor Bage if visit!=6, asis
if _rc==0 {
        predict num_cen if e(sample), pr
        replace num_cen=1-num_cen
        replace num_cen=1 if visit==6
}
else {
        gen num_cen=1
}

gen stab=num_cen/denom_cen
sort id visit
```

```
by id: gen stab2=stab[_n-1] if _n!=1
recode stab2 .=1
drop stab
rename stab2 stab

*Create all the rows that are needed for all s & t combinations
rename visit t
by id: gen N=_N
gen expand=N-t+1
qui expand expand, gen(duplicate)
drop expand

*Generate s and Sort Data
bysort id t: gen s=N-_n+1
gsort id s -t

*Sort Out Censoring Weights*
by id s: gen stab2=stab[_n-1] if _n!=1
replace stab2=1 if stab2==.
by id s: replace stab2=stab2*stab2[_n-1] if _n!=1

*Need FEV1 at Visit S for all t
gen Ys=fev1 if s==t
by id s: replace Ys=Ys[_n-1] if _n!=1

*Create Z & Z interactions with Dnase & Propensity Score
*Create space to store Phi estimates
qui forv n=1/5 {
        gen z`n'=s==t+`n'-1
        gen dnase_z`n'=dnase*z`n'
        gen int_z`n'=dnase*Lfev1*z`n'
        gen ps_z`n'=ps*z`n'
        gen ps_int_z`n'=ps*Lfev1*z`n'
        gen phi`n'=.
        gen phi_int`n'=.
}

*First Iteration Can only Fit Short-Term Model
*i.e. s==t
gen H=Ys if s==t

gen smt=s-t

*Loop through seven times, each time can estimate an addition Phi
qui forv n=1/5 {

        *Get Estimate
        regress H i.dnase_z* int_z* ps_z* ps_int_z* c.Lcum_dnase##i.smt ///
                c.Lcum_dnase#c.Lfev1##i.smt c.Lfev1##i.smt c.Bage##i.smt ///
                i.exac##i.smt c.iv_days##i.smt if s<t+`n' [pw=stab2]
```

```
        forv m=1/5 {
                capture noisily replace phi`m'=_b[1.dnase_z`m'] if `m'<=`n'
                capture noisily replace phi_int`m'=_b[int_z`m'] if `m'<=`n'
        }

        *Create counterfactuals
        qui replace H=Ys if s==t
        qui replace H=H[_n-1] - phi1*dnase_z1[_n-1] - phi_int1*int_z1[_n-1] ///
        if s==t+1
        qui replace H=H[_n-1] - phi1*dnase_z1[_n-1] - phi_int1*int_z1[_n-1] ///
                - phi2*dnase_z2[_n-1] - phi_int2*int_z2[_n-1] if s==t+2
        qui replace H=H[_n-1] - phi1*dnase_z1[_n-1] - phi_int1*int_z1[_n-1] ///
                - phi2*dnase_z2[_n-1] - phi_int2*int_z2[_n-1] ///
                - phi3*dnase_z3[_n-1] - phi_int3*int_z3[_n-1] if s==t+3
        qui replace H=H[_n-1] - phi1*dnase_z1[_n-1] - phi_int1*int_z1[_n-1] ///
                - phi2*dnase_z2[_n-1] - phi_int2*int_z2[_n-1] ///
                - phi3*dnase_z3[_n-1] - phi_int3*int_z3[_n-1] ///
                - phi4*dnase_z4[_n-1] - phi_int4*int_z4[_n-1] if s==t+4
        qui replace H=H[_n-1] - phi1*dnase_z1[_n-1] - phi_int1*int_z1[_n-1] ///
                - phi2*dnase_z2[_n-1] - phi_int2*int_z2[_n-1] ///
                - phi3*dnase_z3[_n-1] - phi_int3*int_z3[_n-1] ///
                - phi4*dnase_z4[_n-1] - phi_int4*int_z4[_n-1] ///
                - phi5*dnase_z5[_n-1] - phi_int5*int_z5[_n-1] if s==t+5
}

*Final Estimates
regress H i.dnase_z* int_z* ps_z* ps_int_z* c.Lcum_dnase##i.smt ///
        c.Lcum_dnase#c.Lfev1##i.smt c.Lfev1##i.smt c.Bage##i.smt i.exac##i.smt ///
        c.iv_days##i.smt [pw=stab2], cluster(id)


*************        Model 10 - G Estimation - No Interactions
*************        IV Days
***************************************************************

rename visit t
by id: gen fut_iv_days=iv_days[_n+1] if _n!=_N

*Propensity Score Model
qui logit dnase i.Ldnase##c.Lfev1 i.Ldnase##i.exac i.Ldnase##c.iv_days Bage
qui predict ps, pr

*Censoring Denominator*
capture noisily logit censor i.dnase fev1 i.exac iv_days Bage if t!=6, asis
if _rc==0 {
        predict denom_cen if e(sample), pr
        replace denom_cen=1-denom_cen
        replace denom_cen=1 if t==6
}
else {
        gen denom_cen=1
```

```
}

*Censoring Numerator*
capture noisily logit censor Bage if t!=6, asis
if _rc==0 {
        predict num_cen if e(sample), pr
        replace num_cen=1-num_cen
        replace num_cen=1 if t==6
}
else {
        gen num_cen=1
}

gen stab=num_cen/denom_cen
drop if t==1 | t==6
sort id t

*Create all the rows that are needed for all s & t combinations
by id: gen N=_N
gen expand=N-t+1
qui expand expand, gen(duplicate)
drop expand duplicate

*Generate s and Sort Data
bysort id t: gen s=N-_n+1
gsort id s -t

*Sort Out Censoring Weights*
by id s: gen stab2=stab[_n-1] if _n!=1
replace stab2=1 if stab2==.
by id s: replace stab2=stab2*stab2[_n-1] if _n!=1

*Need Future IV Days at Visit S for all t
gen Ys=fut_iv_days if s==t
by id s: replace Ys=Ys[_n-1] if _n!=1

*Create Z & Z interactions
*Create space to store Phi estimates
qui forv n=1/4 {
        gen z`n'=s>=t+`n'-1
        gen dnase_z`n'=dnase*z`n'
        gen p_z`n'=ps*z`n'
        gen psi`n'=.
}

*First Iteration Can only Fit Short-Term Model
*i.e. s==t
gen smt=s-t
gen H=Ys if s==t

*Loop through seven times, each time can estimate an addition Phi
```

```
qui forv n=1/4 {
        *Get Estimate
        glm H i.dnase_z? p_z? c.Lcum_dnase##i.smt c.Lfev1##i.smt ///
                c.Bage##i.smt i.exac##i.smt c.iv_days##i.smt if s<t+`n' ///
                [pw=stab2], family(gamma) link(log) scale(1)
        forv m=1/4 {
                capture noisily replace psi`m'=_b[1.dnase_z`m']
        }
        *Create counterfactuals
        replace H=H[_n-1]*exp(-psi1*dnase_z1[_n-1]) if s==t+1
        replace H=H[_n-1]*exp(-psi1*dnase_z1[_n-1]-psi2*dnase_z2[_n-1]) if s==t+2
        replace H=H[_n-1]*exp(-psi1*dnase_z1[_n-1]-psi2*dnase_z2[_n-1] ///
                -psi3*dnase_z3[_n-1]) if s==t+3
        replace H=H[_n-1]*exp(-psi1*dnase_z1[_n-1]-psi2*dnase_z2[_n-1] ///
                -psi3*dnase_z3[_n-1]-psi4*dnase_z4[_n-1]) if s==t+4
        replace H=365 if H>365 & H!=.
}
glm H i.dnase_z? p_z? c.Lcum_dnase##i.smt c.Lfev1##i.smt c.Bage##i.smt ///
        i.exac##i.smt c.iv_days##i.smt [pw=stab2], family(gamma) link(log) ///
        scale(1)

*************        Model 11 - G Estimation - With Interactions
*************        IV Days
****************************************************************

rename visit t
by id: gen fut_iv_days=iv_days[_n+1] if _n!=_N

*Propensity Score Model
logit dnase i.Ldnase##c.Lfev1 i.Ldnase##i.exac i.Ldnase##c.iv_days Bage
qui predict ps, pr

*Censoring Denominator*
capture noisily logit censor i.dnase fev1 i.exac iv_days Bage if t!=6, asis
if _rc==0 {
        predict denom_cen if e(sample), pr
        replace denom_cen=1-denom_cen
        replace denom_cen=1 if t==6
}
else {
        gen denom_cen=1
}

*Censoring Numerator*
capture noisily logit censor Bage if t!=6, asis
if _rc==0 {
        predict num_cen if e(sample), pr
        replace num_cen=1-num_cen
        replace num_cen=1 if t==6
}
else {
```

```
        gen num_cen=1
}

gen stab=num_cen/denom_cen
drop if t==1 | t==6
sort id t

*Create all the rows that are needed for all s & t combinations
by id: gen N=_N
gen expand=N-t+1
qui expand expand, gen(duplicate)
drop expand duplicate

*Generate s and Sort Data
bysort id t: gen s=N-_n+1
gsort id s -t

*Sort Out Censoring Weights*
by id s: gen stab2=stab[_n-1] if _n!=1
replace stab2=1 if stab2==.
by id s: replace stab2=stab2*stab2[_n-1] if _n!=1

*Need Future IV Days at Visit S for all t
gen Ys=fut_iv_days if s==t
by id s: replace Ys=Ys[_n-1] if _n!=1

*Create Z & Z interactions
*Create space to store Phi estimates
qui forv n=1/4 {
        gen z`n'=s>=t+`n'-1
        gen dnase_z`n'=dnase*z`n'
        gen p_z`n'=ps*z`n'
        gen int_iv_z`n'=dnase*iv_days*z`n'
        gen int_exac_z`n'=dnase*exac*z`n'
        gen p_int_z`n'=ps*Lfev1*z`n'
        gen p_int_iv_z`n'=ps*iv_days*z`n'
        gen p_int_exac_z`n'=ps*exac*z`n'

        gen psi`n'=.
        gen psi_iv`n'=.
        gen psi_exac`n'=.
}

*First Iteration Can only Fit Short-Term Model
*i.e. s==t
gen smt=s-t
gen H=Ys if s==t

*Loop through seven times, each time can estimate an addition Phi
qui forv n=1/4 {
        *Get Estimate
```

```
        glm H i.dnase_z? int_iv_z? i.int_exac_z? p_z? p_int_iv_z? ///
                p_int_exac_z? c.Lcum_dnase##i.smt c.Lcum_dnase#c.iv_days##i.smt ///
                c.Lcum_dnase#i.exac##i.smt c.Lfev1##i.smt c.Bage##i.smt ///
                i.exac##i.smt c.iv_days##i.smt if s<t+‘n’ [pw=stab2], ///
                family(gamma) link(log) scale(1)
        forv m=1/4 {
                capture noisily replace psi‘m’=_b[1.dnase_z‘m’]
                capture noisily replace psi_iv‘m’=_b[int_iv_z‘m’]
                capture noisily replace psi_exac‘m’=_b[1.int_exac_z‘m’]
        }
        *Create counterfactuals
        replace H=H[_n-1]*exp(-psi1*dnase_z1[_n-1]-psi_iv1*int_iv_z1[_n-1] ///
                -psi_exac1*int_exac_z1[_n-1]) if s==t+1
        replace H=H[_n-1]*exp(-psi1*dnase_z1[_n-1]-psi_iv1*int_iv_z1[_n-1] ///
                -psi_exac1*int_exac_z1[_n-1]-psi2*dnase_z2[_n-1] ///
                -psi_iv2*int_iv_z2[_n-1]-psi_exac2*int_exac_z2[_n-1]) if s==t+2
        replace H=H[_n-1]*exp(-psi1*dnase_z1[_n-1]-psi_iv1*int_iv_z1[_n-1] ///
                -psi_exac1*int_exac_z1[_n-1]-psi2*dnase_z2[_n-1] ///
                -psi_iv2*int_iv_z2[_n-1]-psi_exac2*int_exac_z2[_n-1] ///
                -psi3*dnase_z3[_n-1]-psi_iv3*int_iv_z3[_n-1] ///
                -psi_exac3*int_exac_z3[_n-1]) if s==t+3
        replace H=H[_n-1]*exp(-psi1*dnase_z1[_n-1]-psi_iv1*int_iv_z1[_n-1] ///
                -psi_exac1*int_exac_z1[_n-1]-psi2*dnase_z2[_n-1] ///
                -psi_iv2*int_iv_z2[_n-1]-psi_exac2*int_exac_z2[_n-1] ///
                -psi3*dnase_z3[_n-1]-psi_iv3*int_iv_z3[_n-1] ///
                -psi_exac3*int_exac_z3[_n-1]-psi4*dnase_z4[_n-1] ///
                -psi_iv4*int_iv_z4[_n-1]-psi_exac4*int_exac_z4[_n-1]) if s==t+4
        replace H=365 if H>365 & H!=.
}
glm H i.dnase_z? int_iv_z? i.int_exac_z? p_z? p_int_iv_z? p_int_exac_z? ///
        c.Lcum_dnase##i.smt c.Lcum_dnase#c.iv_days##i.smt ///
        c.Lcum_dnase#i.exac##i.smt c.Lfev1##i.smt c.Bage##i.smt i.exac##i.smt ///
        c.iv_days##i.smt [pw=stab2], family(gamma) link(log) scale(1)
```

A.3 Tables of Results from Simulation Studies

The following four tables give more detailed results from the simulation studies. The tables correspond to Figures 4 to 9 of the main text.

Scenario	Method	n	1 Year Treatment Effect					5 Year Treatment Effect				
			$\overline{\beta_1}$	Bias	Empirical SE	Model SE	MSE	$\overline{\beta_5}$	Bias	Empirical SE	Model SE	MSE
Standard $\beta_1 = 4.00$ $\beta_5 = 20.00$	IPW of MSM	1000	3.94	-0.06	0.20	0.39	0.04	19.82	-0.18	0.87	1.05	0.79
	IPW of MSM (truncated)	1000	3.63	-0.37	0.20	0.38	0.18	18.66	-1.34	0.86	1.01	2.53
	HA-MSM	1000	4.02	-0.02	0.21	0.21	0.04	20.13	0.13	0.70	0.73	0.50
	HA-MSM (truncated)	1000	3.99	-0.01	0.21	0.21	0.04	19.81	-0.19	0.68	0.72	0.50
	SCMM	1000	3.97	-0.03	0.14	0.14	0.02	NA	NA	NA	NA	NA
	G-Formula	1000	3.96	-0.04	0.15	0.15	0.02	19.62	-0.38	0.58	0.33	0.49
	G-Estimation	1000	3.97	-0.03	0.14	0.14	0.02	20.12	0.12	0.69	1.19	0.49
No Effect $\beta_1 = 0.00$ $\beta_5 = 0.00$	IPW of MSM	1000	-0.02	-0.02	0.21	0.39	0.04	0.05	0.05	0.92	1.08	0.85
	IPW of MSM (truncated)	1000	-0.32	-0.32	0.21	0.38	0.14	-1.09	-1.09	0.90	1.04	2.01
	HA-MSM	1000	-0.01	-0.01	0.22	0.22	0.05	-0.03	-0.03	0.72	0.73	0.53
	HA-MSM (truncated)	1000	-0.01	-0.01	0.21	0.22	0.05	-0.35	0.72	0.72	0.72	0.64
	SCMM	1000	-0.01	-0.01	0.15	0.14	0.02	NA	NA	NA	NA	NA
	G-Formula	1000	-0.01	-0.01	0.16	0.15	0.03	-0.01	-0.01	0.61	0.33	0.37
	G-Estimation	1000	-0.01	-0.01	0.15	0.14	0.02	-0.01	-0.01	0.75	1.22	0.56
Decreasing Effect $\beta_1 = 3.50$ $\beta_5 = 13.03$	IPW of MSM	1000	3.46	-0.04	0.21	0.39	0.05	13.02	-0.01	0.89	1.05	0.79
	IPW of MSM (truncated)	1000	3.15	-0.35	0.21	0.38	0.16	11.87	-1.16	0.88	1.01	2.12
	HA-MSM	1000	3.42	-0.08	0.21	0.22	0.05	13.51	0.48	0.71	0.73	0.73
	HA-MSM (truncated)	1000	3.40	-0.10	0.21	0.21	0.05	13.18	0.15	0.70	0.72	0.51
	SCMM	1000	3.48	-0.02	0.14	0.14	0.05	NA	NA	NA	NA	NA
	G-Formula	1000	3.48	-0.02	0.16	0.15	0.03	12.76	-0.27	0.61	0.33	0.44
	G-Estimation	1000	3.48	-0.02	0.14	0.14	0.05	13.12	0.09	0.72	1.19	0.53
Effect Modification $\beta_1 = 1.97$ $\beta_5 = 9.34$	IPW of MSM	1000	1.96	-0.008	0.21	0.38	0.043	9.34	-0.000	0.77	0.90	0.59
	IPW of MSM (truncated)	1000	1.65	-0.32	0.21	0.37	0.14	8.34	-1.00	0.76	0.86	1.58
	HA-MSM	1000	1.94	-0.029	0.20	0.22	0.041	10.31	0.97	0.67	0.67	1.39
	HA-MSM (truncated)	1000	1.91	-0.057	0.20	0.21	0.043	9.90	0.56	0.66	0.66	0.76
	SCMM	1000	1.97	-0.004	0.15	0.14	0.022	NA	NA	NA	NA	NA
	G-Formula	1000	1.92	-0.05	0.15	0.14	0.026	9.27	-0.067	0.57	0.30	0.33
	G-Estimation	1000	1.97	-0.004	0.15	0.14	0.022	10.03	0.69	0.67	1.12	0.92
Reversed Causal Pathway $\beta_1 = 4.67$ $\beta_5 = 20.38$	IPW of MSM	942	-31.45	-36.12	19.36	3.37	1679.12	32.14	11.76	20.64	5.01	563.79
	IPW of MSM (truncated)	1000	4.85	0.19	0.24	0.43	0.09	19.03	-1.35	0.99	1.15	2.80
	HA-MSM	926	-4.62	-9.29	10.18	2.54	189.74	23.44	3.06	8.19	2.97	76.34
	HA-MSM (truncated)	1000	4.65	-0.02	0.21	0.22	0.04	19.09	-1.29	0.71	0.73	2.16
	SCMM	1000	4.00	-0.67	0.15	0.14	0.46	NA	NA	NA	NA	NA
	G-Formula	1000	4.00	-0.67	0.16	0.15	0.47	18.25	-2.13	0.58	0.32	4.89
	G-Estimation	1000	4.00	-0.67	0.15	0.14	0.46	18.18	-2.20	0.71	1.23	5.36
Censoring $\beta_1 = 4.00$ $\beta_5 = 20.00$	IPW of MSM	1000	3.94	-0.06	0.23	0.45	0.06	19.20	-0.80	1.49	1.59	2.86
	IPW of MSM (truncated)	1000	3.63	-0.37	0.23	0.43	0.19	17.94	-2.06	1.46	6.38	1.54
	HA-MSM	1000	4.02	0.02	0.24	0.25	0.06	19.69	-0.31	1.17	1.46	1.16
	HA-MSM (truncated)	1000	3.99	-0.01	0.24	0.06	0.25	19.27	-0.73	1.15	1.14	1.87
	SCMM	1000	3.97	-0.03	0.16	0.17	0.03	NA	NA	NA	NA	NA
	G-Formula	1000	3.96	-0.04	0.18	0.17	0.03	19.33	-0.67	0.76	0.52	1.02
	G-Estimation	1000	3.97	-0.03	0.16	0.17	0.03	19.70	-0.30	1.10	1.68	1.31

TABLE A3 Simulation study results of population-average effect for continuous outcome ($\overline{X_t} \rightarrow F_t$). NA signifies that the method does not estimate that effect.

Scenario	Method	n	1 Year Treatment Effect					5 Year Treatment Effect				
			$\overline{\beta_1}$	Bias	Empirical SE	Model SE	MSE	$\overline{\beta_5}$	Bias	Empirical SE	Model SE	MSE
No Effect $\beta_1 = 0.00$ $\beta_5 = 0.00$	HA-MSM	1000	0.003	0.003	0.088	0.087	0.008	0.088	0.088	0.29	0.30	0.092
	HA-MSM (truncated)	1000	0.004	0.004	0.086	0.085	0.007	0.19	0.19	0.29	0.30	0.12
	SCMM	1000	-0.000	-0.000	0.060	0.060	0.004	NA	NA	NA	NA	NA
	G-Estimation	1000	-0.000	-0.000	0.060	0.060	0.004	0.012	0.012	0.31	0.026	0.098
Standard (no effect modification) $\beta_1 = 0.00$ $\beta_5 = 0.00$	HA-MSM	1000	-0.009	-0.009	0.084	0.085	0.007	-0.63	-0.63	0.30	0.30	0.48
	HA-MSM (truncated)	1000	-0.004	-0.004	0.081	0.083	0.007	-0.52	-0.52	0.29	0.30	0.35
	SCMM	1000	0.018	0.018	0.054	0.058	0.003	NA	NA	NA	NA	NA
	G-Estimation	1000	0.018	0.018	0.054	0.058	0.003	-0.78	-0.78	0.32	0.025	0.71
Effect Modification $\beta_1 = -0.80$ $\beta_5 = -3.50$	HA-MSM	1000	-0.75	0.050	0.085	0.087	0.010	-3.06	0.44	0.27	0.27	0.27
	HA-MSM (truncated)	1000	-0.74	0.056	0.084	0.085	0.019	-2.95	0.55	0.27	0.27	0.38
	SCMM	1000	-0.78	0.022	0.061	0.060	0.004	NA	NA	NA	NA	NA
	G-Estimation	1000	-0.78	0.022	0.061	0.060	0.004	-3.44	0.064	0.30	0.018	0.092

TABLE A4 Simulation study results of interaction effect for continuous outcome. Results show change in effect of $\overline{X_t}$ on F_t per 10 change in F_{t-1}. NA signifies that the method does not estimate that effect.

Scenario	Estimate	Method	n	1 Year Treatment Effect						4 Year Treatment Effect					
				Log Odds Ratio of Zero Count			Log Rate Ratio of Count			Log Odds Ratio of Zero Count			Log Rate Ratio of Count		
				$\overline{\beta}$	Empirical SE	Model SE	$\overline{\beta}$	Empirical SE	Model SE	$\overline{\beta}$	Empirical SE	Model SE	$\overline{\beta}$	Empirical SE	Model SE
No Effect	Conditional	HA-MSM	1000	-0.002	0.027	0.028	-0.001	0.007	0.007	-0.002	0.055	0.053	0.009	0.026	0.024
		HA-MSM (truncated)	1000	-0.002	0.027	0.028	-0.000	0.006	0.006	-0.006	0.054	0.053	0.012	0.024	0.023
		SCMM	1000	-0.003	0.057	0.056	-0.001	0.007	0.007	NA	NA	NA	NA	NA	NA
		G-Estimation	1000	NA	NA	NA	-0.001	0.010	0.011	NA	NA	NA	-0.000	0.021	0.021
Standard (no effect modification)	Conditional	HA-MSM	1000	-0.008	0.030	0.029	-0.002	0.009	0.008	0.093	0.73	0.071	-0.043	0.054	0.038
		HA-MSM (truncated)	1000	-0.006	0.029	0.028	-0.001	0.008	0.008	0.089	0.073	0.070	-0.040	0.054	0.037
		SCMM	1000	0.001	0.050	0.049	0.002	0.009	0.008	NA	NA	NA	NA	NA	NA
		G-Estimation	1000	NA	NA	NA	0.18	0.020	0.012	NA	NA	NA	-0.033	0.078	0.023
Effect Modification	Conditional	HA-MSM	1000	0.11	0.028	0.028	-0.16	0.017	0.013	0.20	0.057	0.056	-0.090	0.034	0.030
		HA-MSM (truncated)	1000	0.11	0.027	0.027	-0.17	0.014	0.012	0.20	0.057	0.056	-0.086	0.032	0.029
		SCMM	1000	0.21	0.045	0.046	-0.20	0.010	0.010	NA	NA	NA	NA	NA	NA
		G-Estimation	1000	NA	NA	NA	-0.12	0.013	0.011	NA	NA	NA	-0.002	0.050	0.022

TABLE A5 Simulation study results of interaction effects for count outcome. Results show change in effect of $\overline{X_t}$ on V_{t+1} per 10 change in V_t. NA signifies that the method does not estimate that effect.

Scenario	Estimate	Method	n	1 Year Treatment Effect						4 Year Treatment Effect					
				Log Odds Ratio of Zero Count			Log Rate Ratio of Count			Log Odds Ratio of Zero Count			Log Rate Ratio of Count		
				$\overline{\beta}$	Empirical SE	Model SE	$\overline{\beta}$	Empirical SE	Model SE	$\overline{\beta}$	Empirical SE	Model SE	$\overline{\beta}$	Empirical SE	Model SE
Standard	Marginal	IPW of MSM	1000	1.28	0.033	0.040	-0.56	0.037	0.038	3.13	0.16	0.16	-1.27	0.15	0.14
		IPW of MSM (truncated)	1000	1.27	0.033	0.039	-0.55	0.037	0.038	3.08	0.16	0.16	-1.22	0.17	0.15
		G-Formula	1000	1.28	0.031	0.016	-0.57	0.026	0.017	3.12	0.11	0.067	-1.27	0.085	0.067
	Conditional	HA-MSM	1000	1.63	0.043	0.045	-0.67	0.023	0.024	3.71	0.17	0.17	-1.39	0.11	0.11
		HA-MSM (truncated)	1000	1.64	0.043	0.045	-0.67	0.023	0.023	3.68	0.17	0.17	-1.37	0.11	0.10
		SCMM	1000	2.08	0.057	0.060	-0.82	0.025	0.024	NA	NA	NA	NA	NA	NA
		G-Estimation	1000	NA	NA	NA	-2.28	0.063	0.016	NA	NA	NA	-4.30	0.19	0.042
No Effect	Marginal	IPW of MSM	1000	-0.001	0.028	0.035	0.003	0.019	0.022	-0.001	0.071	0.075	0.006	0.047	0.053
		IPW of MSM (truncated)	1000	-0.011	0.028	0.035	0.014	0.018	0.020	-0.042	0.071	0.074	0.042	0.045	0.048
		G-Formula	1000	-0.001	0.026	0.012	0.002	0.015	0.008	-0.002	0.056	0.027	0.001	0.030	0.018
	Conditional	HA-MSM	1000	-0.001	0.037	0.039	0.001	0.015	0.016	-0.002	0.073	0.074	0.004	0.039	0.041
		HA-MSM (truncated)	1000	0.000	0.037	0.039	0.001	0.015	0.016	-0.012	0.073	0.074	0.013	0.038	0.039
		SCMM	1000	-0.001	0.047	0.048	0.000	0.016	0.017	NA	NA	NA	NA	NA	NA
		G-Estimation	1000	NA	NA	NA	0.002	0.030	0.016	NA	NA	NA	0.001	0.058	0.042
Decreasing Effect	Marginal	IPW of MSM	1000	1.12	0.032	0.038	-0.49	0.033	0.034	1.89	0.098	0.10	-0.79	0.074	0.076
		IPW of MSM (truncated)	1000	1.11	0.033	0.038	-0.48	0.033	0.033	1.84	0.097	0.099	-0.75	0.074	0.074
		G-Formula	1000	1.12	0.029	0.015	-0.50	0.022	0.015	1.88	0.077	0.040	-0.78	0.048	0.031
	Conditional	HA-MSM	1000	1.39	0.041	0.042	-0.55	0.021	0.022	2.37	0.11	0.11	-0.92	0.065	0.063
		HA-MSM (truncated)	1000	1.40	0.041	0.042	-0.56	0.021	0.022	2.35	0.11	0.11	-0.90	0.062	0.062
		SCMM	1000	1.82	0.057	0.055	-0.72	0.022	0.022	NA	NA	NA	NA	NA	NA
		G-Estimation	1000	NA	NA	NA	-1.94	0.055	0.016	NA	NA	NA	-2.46	0.12	0.042
Effect Modification	Marginal	IPW of MSM	1000	0.92	0.034	0.039	-0.96	0.030	0.031	1.75	0.097	0.098	-0.81	0.065	0.066
		IPW of MSM (truncated)	1000	0.91	0.034	0.039	-0.95	0.028	0.029	1.71	0.096	0.097	-0.78	0.058	0.060
		G-Formula	1000	0.92	0.030	0.014	-0.72	0.023	0.010	1.68	0.076	0.039	-0.56	0.042	0.025
	Conditional	HA-MSM	1000	1.11	0.041	0.042	-0.75	0.026	0.027	2.08	0.10	0.10	-0.82	0.060	0.058
		HA-MSM (truncated)	1000	1.11	0.041	0.042	-0.076	0.026	0.026	2.06	0.10	0.10	-0.81	0.058	0.057
		SCMM	1000	1.33	0.052	0.052	-0.84	0.028	0.029	NA	NA	NA	NA	NA	NA
		G-Estimation	1000	NA	NA	NA	-1.24	0.049	0.016	NA	NA	NA	-1.87	0.096	0.042
Reversed Causal Pathway	Marginal	IPW of MSM	758	-2.09	2.71	0.48	0.96	0.86	0.10	10.69	60.95	1.76	-0.93	12.37	0.18
		IPW of MSM (truncated)	1000	1.21	0.051	0.058	-0.37	0.088	0.089	2.21	0.16	0.16	-0.76	0.13	0.12
		G-Formula	1000	1.06	0.042	0.017	-0.41	0.033	0.014	2.16	0.11	0.057	-0.74	0.069	0.042
	Conditional	HA-MSM	727	1.19	0.29	0.14	-0.11	0.50	0.37	3.13	2.19	0.65	-0.83	0.68	0.69
		HA-MSM (truncated)	1000	1.35	0.049	0.049	-0.47	0.032	0.032	2.44	0.15	0.15	-0.81	0.11	0.11
		SCMM	1000	1.42	0.060	0.061	-0.54	0.034	0.036	NA	NA	NA	NA	NA	NA
		G-Estimation	1000	NA	NA	NA	-1.72	0.071	0.016	NA	NA	NA	-2.63	0.16	0.043
Censoring	Marginal	IPW of MSM	1000	1.30	0.044	0.050	-0.60	0.033	0.034	3.14	0.28	0.28	-1.21	0.19	0.18
		IPW of MSM (truncated)	1000	1.27	0.043	0.049	-0.59	0.032	0.033	3.01	0.27	0.27	-1.17	0.19	0.17
		G-Formula	1000	1.32	0.042	0.020	-0.60	0.029	0.017	3.12	0.18	0.11	-1.25	0.11	0.080
	Conditional	HA-MSM	1000	1.70	0.059	0.059	-0.68	0.031	0.030	3.68	0.28	0.29	-1.32	0.18	0.17
		HA-MSM (truncated)	1000	1.71	0.059	0.058	-0.69	0.029	0.028	0.28	0.28	0.28	-1.30	0.18	0.17
		SCMM	1000	2.08	0.082	0.081	-0.81	0.034	0.034	NA	NA	NA	NA	NA	NA
		G-Estimation	1000	NA	NA	NA	-2.29	0.083	0.082	NA	NA	NA	-4.32	0.31	0.32

TABLE A6 Simulation study results of population-average effects for count outcome ($\overline{X_t} \rightarrow V_{t+1}$). NA signifies that the method does not estimate that effect.

Method	1 Year Treatment Effect			2 Year Treatment Effect			3 Year Treatment Effect			4 Year Treatment Effect			5 Year Treatment Effect		
	coef.	95% CI	P	coef.	95% CI	P	coef.	95% CI	P	coef.	95% CI	P	coef.	95% CI	P
IPW of MSM	-3.15	-4.21, -2.08	<0.001	-4.69	-5.98, -3.96	<0.001	-6.04	-7.38, -4.71	<0.001	-7.39	-8.94, -5.84	<0.001	-8.49	-10.34, -6.65	<0.001
IPW of MSM (truncated)	-3.22	-4.24, -2.21	<0.001	-4.56	-5.65, -3.48	<0.001	-6.23	-7.47, -4.98	<0.001	-7.63	-9.08, -6.19	<0.001	-8.81	-10.50, -7.12	<0.001
HA-MSM	-1.06	-1.79, -0.32	0.005	-0.81	-1.56, -0.066	0.033	-1.01	-1.98, -0.044	0.040	-1.13	-2.35, 0.091	0.070	-1.52	-3.30, 0.27	0.097
HA-MSM (truncated)	-0.94	-1.61, -0.27	0.006	-0.88	-1.61, -0.15	0.018	-1.10	-2.03, -0.16	0.022	-1.23	-2.39, -0.060	0.39	-1.82	-3.44, -0.21	0.027
SCMM	-0.041	-0.50, 0.42	0.86	NA	NA	NA	NA	NA	NA	NA	NA	NA	NA	NA	NA
G-Formula	0.035	-0.47, 0.54	0.89	-0.89	-1.64, -0.14	0.020	-2.09	-2.98, 01.19	<0.001	-3.30	-4.40, -2.21	<0.001	-5.16	-6.45, -3.87	<0.001
G-Estimation	-0.041	-0.50, 0.42	0.86	-0.57	-1.26, 0.13	0.11	-1.06	-2.00, -0.11	0.028	-1.53	-2.77, -0.30	0.015	-2.03	-3.56, -0.50	0.009

TABLE A7 Data analysis results of population-average effect of $\overline{X_t}$ on F_t (a continuous outcome). NA signifies that the method does not estimate that effect.

Method	Term	1 Year Treatment Effect			2 Year Treatment Effect			3 Year Treatment Effect			4 Year Treatment Effect			5 Year Treatment Effect		
		coef.	95% CI	P	coef.	95% CI	P	coef.	95% CI	P	coef.	95% CI	P	coef.	95% CI	P
HA-MSM	Intercept	3.32	0.22, 6.41	0.036	2.11	-1.03, 5.25	0.19	2.34	-1.79, 6.47	0.27	4.50	-1.21, 10.21	0.12	8.30	-0.45, 17.05	0.063
	Interaction	-0.57	-0.95, -0.19	0.004	-0.37	-0.76, 0.017	0.061	-0.43	-0.94, 0.087	0.10	-0.73	-1.44, -0.019	0.044	-1.29	-2.36, -0.22	0.018
HA-MSM (truncated)	Intercept	3.32	0.48, 6.16	0.022	2.02	-1.02, 5.06	0.19	2.39	-1.52, 6.31	0.23	4.56	-0.91, 10.02	0.10	7.74	0.47, 15.02	0.037
	Interaction	-0.55	-0.90, -0.20	0.002	-0.37	-0.75, 0.011	0.057	-0.45	-0.94, 0.043	0.074	-0.75	-1.44, -0.065	0.032	-1.26	-2.16, -0.35	0.006
SCMM	Intercept	2.71	0.078, 5.35	0.044	NA	NA	NA	NA	NA	NA	NA	NA	NA	NA	NA	NA
	Interaction	-0.37	-0.72, -0.019	0.039	NA	NA	NA	NA	NA	NA	NA	NA	NA	NA	NA	NA
G-Estimation	Intercept	2.71	0.078, 5.35	0.044	4.85	-1.97, 11.68	0.16	5.12	-7.08, 17.33	0.41	8.54	-10.93, 28.02	0.39	16.12	-13.41, 45.64	0.28
	Interaction	-0.37	-0.72, -0.019	0.039	-0.70	-1.59, 0.18	0.12	-0.86	-2.43, 0.72	0.29	-1.52	-4.01, 0.98	0.23	-2.69	-6.46, 1.07	0.16

TABLE A8 Data analysis results including effect modification for effect of $\overline{X_t}$ on F_t. The intercept term is the estimated effect for an individual with $F_{t-1} = 0$, and the interaction effect is the estimated change per 10 increase in F_{t-1}. NA signifies that the method does not estimate that effect.

Method	Term	1 Year Treatment Effect			2 Year Treatment Effect			3 Year Treatment Effect			4 Year Treatment Effect		
		OR or RR	95% CI	P	OR or RR	95% CI	P	OR or RR	95% CI	P	OR or RR	95% CI	P
IPW of MSM	Odds of Zero	0.66	0.58, 0.75	<0.001	0.61	0.54, 0.69	<0.001	0.60	0.52, 0.69	<0.001	0.55	0.46, 0.66	<0.001
	Rate of Count	1.15	1.04, 1.27	0.009	1.15	1.05, 1.26	0.002	1.15	1.05, 1.27	0.004	1.19	1.05, 1.34	0.005
IPW of MSM (truncated)	Odds of Zero	0.66	0.59, 0.75	<0.001	0.61	0.54, 0.69	<0.001	0.60	0.52, 0.69	<0.001	0.54	0.45, 0.65	<0.001
	Rate of Count	1.15	1.05, 1.26	0.002	1.16	1.07, 1.26	<0.001	1.17	1.07, 1.28	<0.001	1.21	1.08, 1.35	0.001
HA-MSM	Odds of Zero	0.71	0.62, 0.81	<0.001	0.79	0.67, 0.92	0.002	0.95	0.79, 1.13	0.55	0.95	0.76, 1.19	0.66
	Rate of Count	1.15	1.07, 1.25	<0.001	1.03	0.97, 1.11	0.33	1.07	0.98, 1.17	0.12	1.09	0.96, 1.23	0.18
HA-MSM (truncated)	Odds of Zero	0.72	0.63, 0.83	<0.001	0.78	0.67, 0.91	0.002	0.93	0.78, 1.11	0.43	0.91	0.73, 1.13	0.41
	Rate of Count	1.14	1.07, 1.22	<0.001	1.04	0.97, 1.11	0.25	1.09	1.00, 1.18	0.044	1.12	1.01, 1.24	0.036
SCMM	Odds of Zero	0.85	0.75, 0.96	0.008	NA	NA	NA	NA	NA	NA	NA	NA	NA
	Rate of Count	1.08	1.03, 1.12	0.001	NA	NA	NA	NA	NA	NA	NA	NA	NA
G-Formula	Odds of Zero	0.89	0.81, 0.98	0.016	0.76	0.68, 0.85	<0.001	0.68	0.59, 0.77	<0.001	0.65	0.56, 0.76	<0.001
	Rate of Count	1.05	1.00, 1.11	0.061	1.08	1.00, 1.15	0.043	1.10	1.01, 1.19	0.038	1.14	1.03, 1.26	0.013
G-Estimation	Rate of Count	1.25	1.15, 1.36	<0.001	1.36	1.21, 1.53	<0.001	1.40	1.21, 1.61	<0.001	1.44	1.23, 1.69	<0.001

TABLE A9 Data analysis results of population-average effect of $\overline{X_t}$ on V_{t+1} (a count outcome). NA signifies that the method does not estimate that effect.

Method	Term		1 Year Treatment Effect			2 Year Treatment Effect			3 Year Treatment Effect			4 Year Treatment Effect		
			OR or RR	95% CI	P	OR or RR	95% CI	P	OR or RR	95% CI	P	OR or RR	95% CI	P
HA-MSM	Odds of Zero	Intercept	0.62	0.53, 0.73	<0.001	0.68	0.57, 0.83	<0.001	0.88	0.72, 1.09	0.24	0.89	0.68, 1.15	0.36
		Interaction	1.16	1.03, 1.31	0.012	1.16	1.03, 1.30	0.015	1.07	0.93, 1.24	0.33	1.08	0.94, 1.26	0.28
	Rate of Count	Intercept	1.18	1.06, 1.31	0.002	1.07	0.99, 1.17	0.096	1.17	1.05, 1.31	0.005	1.19	1.03, 1.37	0.017
		Interaction	0.99	0.97, 1.01	0.23	0.98	0.96, 1.01	0.13	0.96	0.93, 0.99	0.014	0.96	0.92, 1.00	0.041
HA-MSM (truncated)	Odds of Zero	Intercept	0.63	0.53, 0.74	<0.001	0.68	0.56, 0.82	<0.001	0.87	0.70, 1.07	0.18	0.85	0.66, 1.10	0.21
		Interaction	1.17	1.04, 1.32	0.007	1.15	1.03, 1.30	0.017	1.07	0.93, 1.23	0.34	1.08	0.93, 1.25	0.30
	Rate of Count	Intercept	1.18	1.07, 1.30	0.001	1.07	0.99, 1.17	0.087	1.18	1.05, 1.32	0.004	1.21	1.05, 1.38	0.007
		Interaction	0.99	0.97, 1.00	0.13	0.98	0.96, 1.01	0.16	0.96	0.94, 0.99	0.019	0.97	0.93, 1.00	0.064
SCMM	Odds of Zero	Intercept	0.73	0.62, 0.86	<0.001	NA	NA	NA	NA	NA	NA	NA	NA	NA
		Interaction	1.16	1.03, 1.30	0.013	NA	NA	NA	NA	NA	NA	NA	NA	NA
	Rate of Count	Intercept	1.12	1.05, 1.20	<0.001	NA	NA	NA	NA	NA	NA	NA	NA	NA
		Interaction	0.98	0.97, 1.00	0.034	NA	NA	NA	NA	NA	NA	NA	NA	NA
G-Estimation	Rate of Count	Intercept	1.36	1.21, 1.52	<0.001	1.68	1.39, 2.02	<0.001	1.94	1.50, 2.53	<0.001	2.29	1.63, 3.24	<0.001
		Interaction	0.94	0.91, 0.97	<0.001	0.94	0.91, 0.97	<0.001	0.96	0.92, 0.99	0.023	0.95	0.91, 1.00	0.037

TABLE A10 Data analysis results including effect modification for effect of $\overline{X_t}$ on V_{t+1}. The intercept term is the estimated effect for an individual with $V_t = 0$, and the interaction effect is the estimated change per 10 increase in V_t. NA signifies that the method does not estimate that effect.
